# Assessment of cerebrovascular alterations induced by inflammatory response and oxidative–nitrative stress after traumatic intracranial hypertension and a potential mitigation strategy

**DOI:** 10.1038/s41598-024-64940-6

**Published:** 2024-06-24

**Authors:** Shangming Zhang, Yehuang Chen, Qizuan Chen, Hongjie Chen, Liangfeng Wei, Shousen Wang

**Affiliations:** 1 Fuzong Clinical Medical College of Fujian Medical University, Fuzhou, 350025 China; 2 Department of Neurosurgery, 900th Hospital, Fujian Provincial Clinical Medical Research Center for Minimally Invasive Diagnosis and Treatment of Neurovascular Diseases, Fuzhou, 350025 China

**Keywords:** Acute subdural hematoma, Cerebral microcirculation, Inflammatory response, Oxidative–nitrative stress, Decompressive craniectomy, Diffuse brain swelling, Blood-brain barrier, Trauma

## Abstract

The rapid perfusion of cerebral arteries leads to a significant increase in intracranial blood volume, exposing patients with traumatic brain injury to the risk of diffuse brain swelling or malignant brain herniation during decompressive craniectomy. The microcirculation and venous system are also involved in this process, but the precise mechanisms remain unclear. A physiological model of extremely high intracranial pressure was created in rats. This development triggered the TNF-α/NF-κB/iNOS axis in microglia, and released many inflammatory factors and reactive oxygen species/reactive nitrogen species, generating an excessive amount of peroxynitrite. Subsequently, the capillary wall cells especially pericytes exhibited severe degeneration and injury, the blood–brain barrier was disrupted, and a large number of blood cells were deposited within the microcirculation, resulting in a significant delay in the recovery of the microcirculation and venous blood flow compared to arterial flow, and this still persisted after decompressive craniectomy. Infliximab is a monoclonal antibody bound to TNF-α that effectively reduces the activity of TNF-α/NF-κB/iNOS axis. Treatment with Infliximab resulted in downregulation of inflammatory and oxidative–nitrative stress related factors, attenuation of capillary wall cells injury, and relative reduction of capillary hemostasis. These improved the delay in recovery of microcirculation and venous blood flow.

## Introduction

Traumatic brain injury (TBI) is a complex multifactorial disease. In addition to the primary injury due to external impact, secondary injuries (e.g., hematoma, cascade reactions in cells, inflammation, and stress injuries) can occur within the first few minutes after injury^1^. Acute subdural hematoma (ASDH) is associated with hemorrhage of the cortical vessels in brain contusions, which can cause extensive compression of the cerebral hemispheres. The hematoma can also cause persistent elevation of intracranial pressure (ICP), leading to diffuse brain swelling (DBS)^2–4^. Decompressive craniectomy (DC) is prone to severe intraoperative brain herniation, thereby decreasing the prognosis of such patients.

Impairment of brainstem vasomotor centers as a result of angular acceleration is the most widely accepted theory for the mechanism of intraoperative brain herniation, highlighting the major role of cerebral arterial blood flow^5,6^. However, the microcirculation and venous system, which contain the highest blood volume in the brain, are also important. Delayed cerebral capillary and venous returns, which play a key role in the development of malignant intraoperative brain herniation, was observed in animal experiments^7^.

The neurovascular unit (NVU) is the minimum functional unit for maintaining the integrity of the blood–brain barrier (BBB) and the homeostatic balance of brain cells^8^. In the NVU, a large number of microglia surround the perivascular spaces of cerebral microvessels, and their end-feet closely communicate with the pericytes and endothelial cells. The capillary diameter and blood flow are closely correlated with signal transduction between microglial end-feet and capillary wall cells, especially pericytes. Recent studies have shown that both denaturation and contraction of pericytes can lead to regional microcirculatory hypoperfusion after TBI, thus inducing microcirculatory disturbance and reperfusion injury^9–11^.

Many studies have demonstrated that pericyte-mediated microcirculatory disturbance are widely affected by neuroinflammation and oxidative–nitrative stress^12,13^. However, microglia are the major immune cells involved in neuroinflammation and oxidative–nitrative stress. Activated microglia can amplify the inflammatory response and induce oxidative–nitrative stress through the release of pro-inflammatory factors and free radicals, which are also the initial markers of secondary injury^8,14^. TNF-α is the most important pro-inflammatory factor secreted by activated microglia and plays a central role in the initiation and regulation of the inflammatory cascade and oxidative–nitrative stress in the NF-κB/iNOS pathway^15,16^. A large number of ROS/RNS products generated by this pathway can induce lipid peroxidation of the cell membrane, resulting in the destruction of phospholipid-dependent enzymes and ion channels, and the loss of function of capillary wall cells, leading to microcirculatory blood flow disturbance, BBB damage and brain swelling. Infliximab (IFX), a monoclonal antibody bound to TNF-α, has anti-inflammatory, anti-oxidative stress and anti-apoptosis effects, and is believed to alleviate the damage caused by hypoxia, ischemia, edema and vascular changes in the acute phase of TBI^17^. TNF-α is involved in various stages of TBI, and blocking TNF-α has been shown to promote neural function recovery after TBI, and is considered as a potential therapeutic target, but its mechanism has not been fully clarified^18,19^.

Therefore, considering the TNF-α/NF-κB/iNOS pathway as an important pathway of neuroinflammatory response and oxidative–nitrative stress, inflammatory response and oxidative–nitrative stress after intracranial hypertension of TBI may induce corresponding cerebral vascular changes, thus inducing the formation of DBS or intraoperative cerebral hernia. Based on this, this study aimed to investigate the mechanism of cerebrovascular and perivascular alterations under acute intracranial hypertension from the perspective of pericytes damage through the rat model of acute subdural hematoma, and further explored potential mitigation strategies.

## Results

### Physiological responses to factors of ASDH and DC

Body weight, baseline mean arterial pressure (MAP), and baseline ICP of the rats were consistent across groups, with no significant differences. None of the rats died during the observational period. Arterial blood gas analysis at the beginning of mechanical ventilation and after observation of the model establishment showed consistent pH, pO2, and pCO2 results. There were no significant differences among and within the groups, and mechanical ventilation did not disrupt the blood gas environment in the rat model (Table 1).

**Table 1:** Comparison of the various indicators in the rat models.

	Sham	ASDH	ASDH + DC	F	P
	(n = 18)	(n = 18)	(n = 18)		
1 Weight(g)	297.75 ± 9.38	295.83 ± 9.44	300.25 ± 11.53	0.570	0.571
2 MAP(mmHg)					
Baseline	90.83 ± 4.28	91.03 ± 3.89	89.57 ± 4.70	0.402	0.672
After modeling	88.72 ± 4.32	110.83 ± 8.52	109.51 ± 8.47	33.990	0.000
3 ICP(mmHg)					
Baseline	4.23 ± 0.50	4.18 ± 0.59	4.15 ± 0.49	0.062	0.940
After modeling	4.40 ± 0.43	46.23 ± 7.51	46.43 ± 8.15	171.532	0.000
4 ABG index					
Baseline					
PH	7.39 ± 0.03	7.41 ± 0.04	7.38 ± 0.03	1.662	0.205
PO2	128.39 ± 7.51	129.70 ± 5.25	127.14 ± 7.53	0.419	0.661
PCO2	38.41 ± 2.62	37.60 ± 2.43	39.39 ± 2.56	1.499	0.238
After modeling					
PH	7.40 ± 0.04	7.40 ± 0.05	7.39 ± 0.05	1.662	0.205
PO2	131.32 ± 6.18	130.07 ± 9.06	130.09 ± 9.40	0.088	0.916
PCO2	37.62 ± 2.42	38.41 ± 3.11	38.63 ± 3.47	0.371	0.693

**Figure 1:**
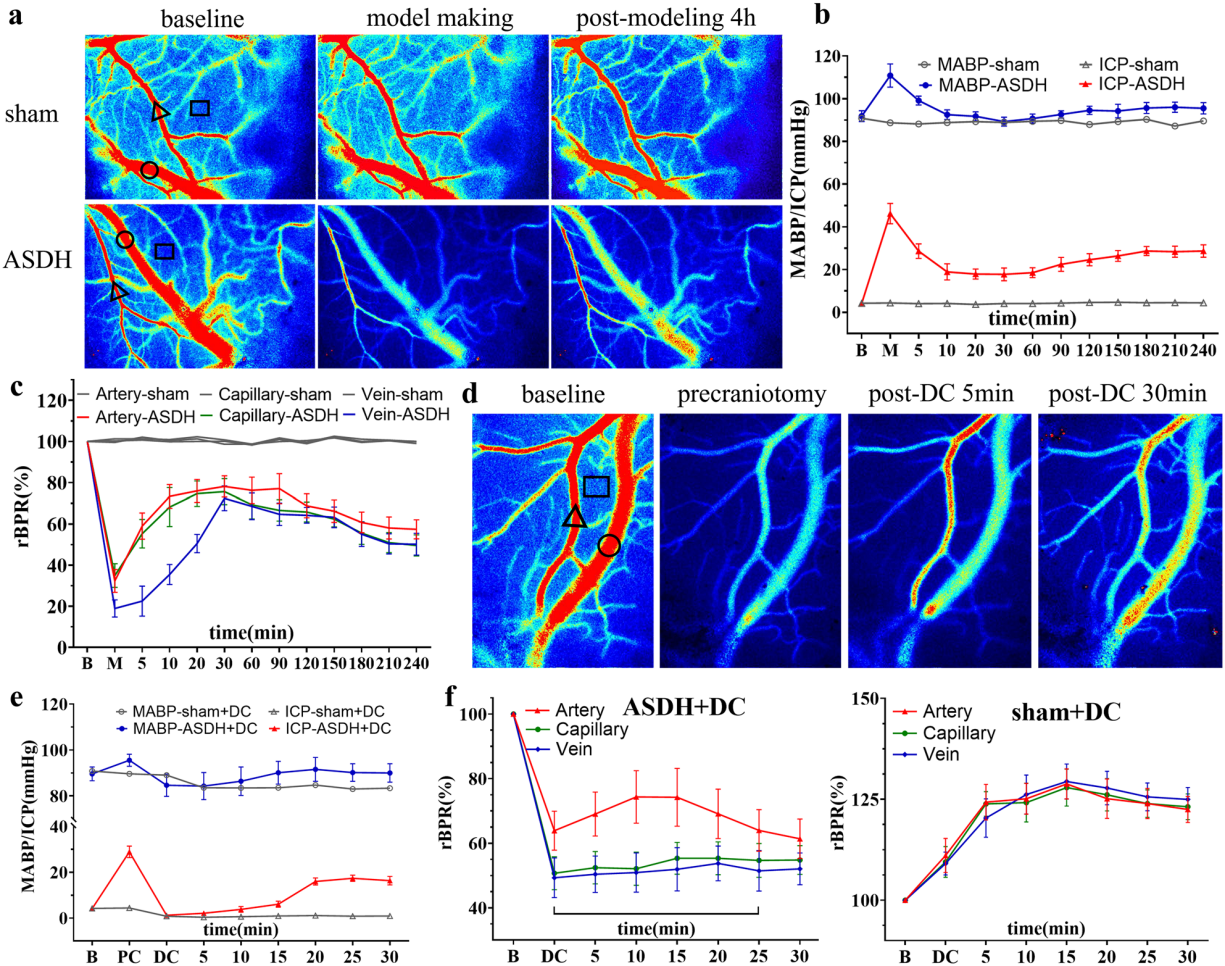
Physiological responses associated with traumatic ASDH and DC in the rat model. (**a**) LSCI monitoring of the ROI of the cerebral cortex on the side without hematoma injection before, immediately after, and 4 h after model establishment. (**b**) Dynamic curve of the changes in baseline MAP and ICP in the sham and ASDH groups. (**c**) Dynamic curve of the changes in rBPR in different vessels of the cerebral cortex on the side without hematoma injection in the sham and ASDH groups. (**d**) LSCI monitoring of the ROI of the cerebral cortex on the hematoma side before and after DC. (**e**) Dynamic curve of the changes in baseline MAP and ICP in the ASDH-DC group after DC. (**f**) Dynamic curve of the changes in rBPR in different vessels of the cerebral cortex on the hematoma side in the ASDH-DC group. Triangles, circles, and squares indicate CBF monitoring sites in arterial, venous, and capillary regions, respectively. Scale bars = 1 mm. B on the X-axis: the baseline value before modeling, M: the highest value in the molding process, PC: the value at 4h after modeling, DC: the highest value during decompressive craniectomy. Values are expressed as the mean ± standard deviation (n = 6 per group).

ASDH factors produced large fluctuations in various physiological data, with transient peak in ICP values at 46.23 ± 7.51 mmHg. At the end of ASDH injection, ICP values decreased and stabilized within 30 min to a mean of 18.26 ± 3.25 mmHg owing to spreading of the hematoma in the subdural space and loss of plasma. This was higher than the mean of the sham group at all time points (p < 0.01). However, brain tissue started to swell at 60–240 min, increasing the ICP up to 28.70 ± 4.56 mmHg (Fig. 1b). Arterial, capillary, and venous cerebral blood flow (CBF) decreased rapidly and synchronously after ASDH injection, with venous vascular segments exhibiting a greater decrease in relative blood perfusion rate (rBPR) after compression than arterioles or capillaries (18.96 ± 6.59 vs. 32.37 ± 9.75 and 34.99 ± 9.07, %, p < 0.05). As ICP decreased, the rBPR in the arterial and capillary segments were higher than those in the venous segment at each time point over the first 30 min. However, as ICP increased again after 60 min, the rBPR of each vascular segment simultaneously decreased again. The rBPR of different vessel types in the ASDH group were significantly different than that in the sham group at all time points (p < 0.01) (Fig. 1a,c). This indicated that ASDH factors increased the ICP and caused extensive compression of cortical blood vessels, resulting in a differential response of various blood vessel types.

After DC and hematoma evacuation, the ICP of rats in the ASDH-DC group decreased rapidly to near 0 and then gradually increased again, reaching 16.34 ± 2.95 mmHg at 30 min. The sham group did not develop intraoperative brain herniation after DC. In the sham group, the rBPR of the vascular segments on the injured side increased rapidly after DC due to the sharp decrease in ICP, reaching 124.8–129.4% of the baseline values and remaining higher than the baseline values at 30 min. In the ASDH-DC group, CBF in the arterial vascular segments increased rapidly after DC and decreased gradually during brain swelling and herniation. In contrast, capillary and venous rBPR exhibited significantly delayed recovery, with neither eventually returning to baseline (Fig. 1d,e). Thus, although DC can cause a rapid decrease in ICP and restore blood supply by filling the arterial vessels in the brain, the delayed response in the capillary and venous segments leads to hyperperfusion of brain tissue and rapid onset of swelling.

### Blood cell deposition in the microvasculature

**Figure 2:**
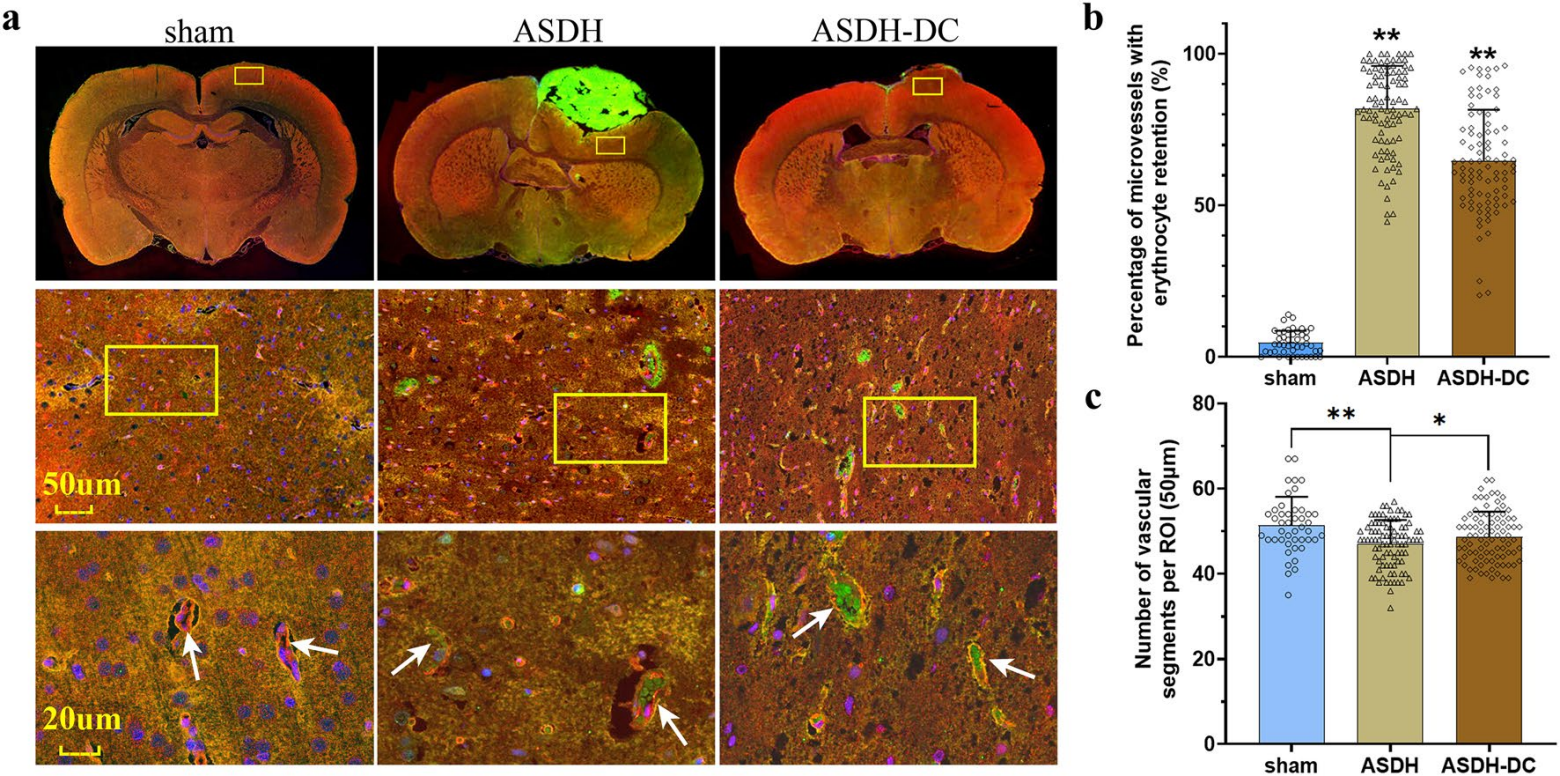
Decelerated blood flow and microvascular blood cell deposition in the rat model of intracranial hypertension. (**a**) IF results showed that a large number of blood cells were deposited in the cortical vascular lumen near the hematoma side after 12 h in the ASDH and ASDH-DC groups. In the 50 um ROI section, the yellow box was randomly selected, and the vascular lumen (white arrow, 20 um) was counted using ZO-1 staining for convenient observation. Pink, vascular wall (ZO-1); green, blood cells; blue, nuclei (DAPI). (**b**) The proportion of blood vessels with red blood cell deposits in the sham, ASDH and ASDH-DC groups. (**c**) The proportion of the total number of blood vessels that could be observed in the ROIs in all groups. In the 50 um ROI section, the yellow box was randomly selected and the vascular lumen (white arrow) was counted using ZO-1 staining for convenient observation. Values are expressed as the mean ± standard deviation (n = 6 per group). *p < 0.05, **p < 0.01.

Blood cell deposition was regional specificity, and the result was more obvious the closer to the hematoma side. We selected cerebral cortex around the thickest part of the hematoma for comparison. In the ASDH group, a large amount of red blood cell deposition and extravasation of red blood cells were observed in the capillaries. In addition, these were still present after DC and did not decrease significantly (Fig. 2a). In immunofluorescent (IF) sections, the number of observable microvessels was calculated by ZO-1 staining, and the number of microvessels with blood cell deposition was calculated by ZO-1 and blood cell co-localization. The co-localization analysis indicated significantly more vessels with blood cell deposition within the 50-µm regions of interest (ROIs) in the ASDH and ASDH-DC groups than in the sham group (p < 0.01) (Fig. 2b). However, no significant difference was found between the ASDH and ASDH-DC groups. With respect to the number of microvessels in the ROIs, the number of observable vessels in the ASDH and ASDH-DC groups was lower than in the sham group (p < 0.01). However, the number of microvessels in the ASDH-DC group was slightly higher than that in the ASDH group (p < 0.05), because some capillaries were restored after the extravascular pressure was relieved (Fig. 2c). The deposition of a large number of red blood cells in the vasculature, along with occlusion of microvessels, directly caused decreased effective CBF during intracranial hypertension, even after DC.

### Vascular wall lesions, microglial activation, inflammatory response and oxidative stress

**Figure 3:**
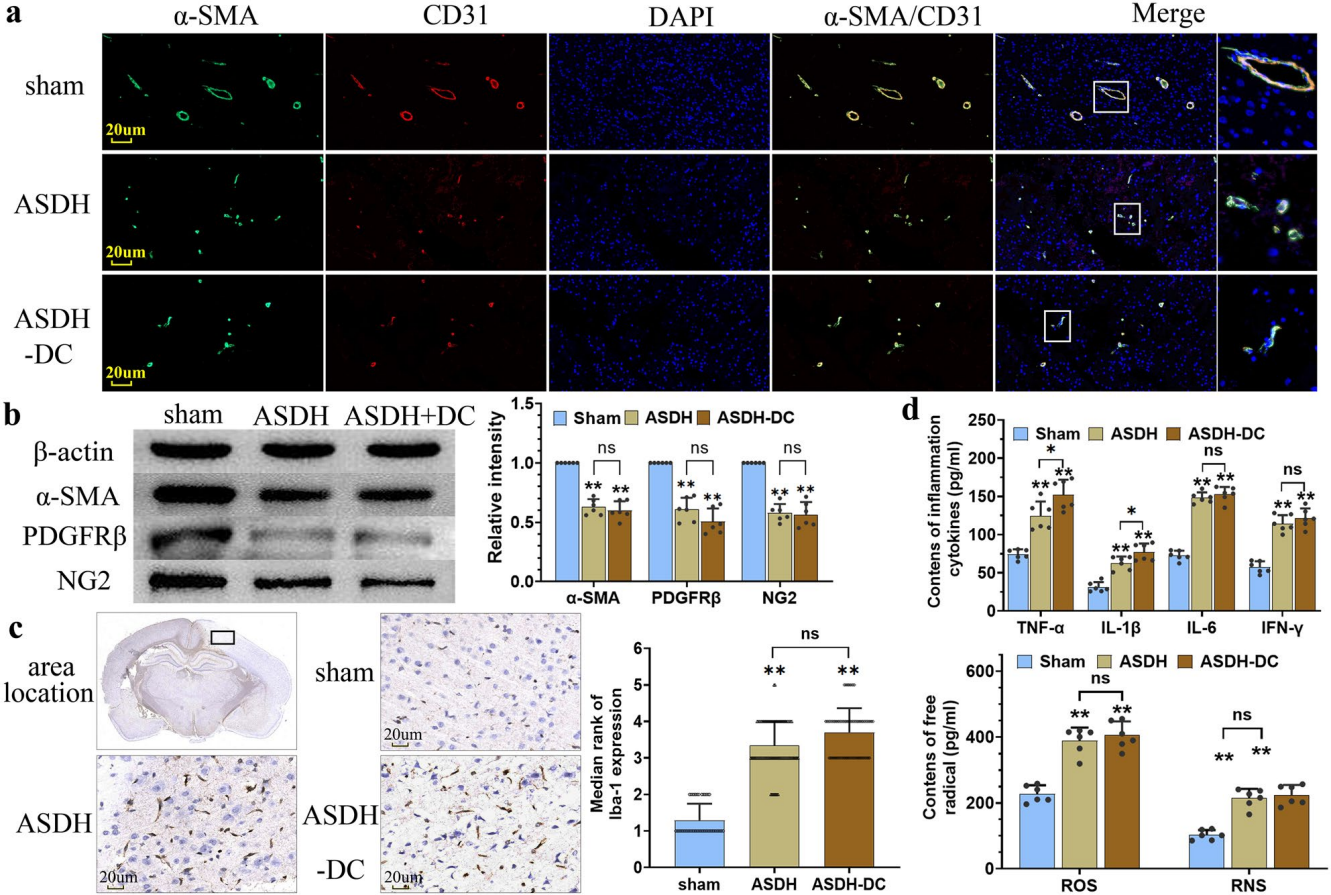
Results of pericyte, endothelial cell, microglial, inflammation factors, and oxidative–nitrative stress factors in rats. (**a**) Representative IF images showed the reduced pericyte and endothelial cell marker staining and severe structural damage to the vascular wall in the ASDH and ASDH-DC groups. (**b**) WB analysis of the expression of the pericyte markers PDGFRβ, α-SMA, and NG2 in the brain tissue near the hematoma side. (**c**) The expression of the microglial marker Iba-1 at 12 h after modeling. The black box was the sample area of superficial cortical tissue taken from the hematoma side. (**d**) ELISA analysis of the production of inflammatory factors (TNF-α, IL-1β, IL-6, and IFN-γ) and free radicals (ROS, RNS) around the injury areas. Values are expressed as the mean ± standard deviation (n = 6 per group), **p < 0.01.

There is still no specific immune marker for pericytes, but many studies have used PDGFRβ, α-SMA, and NG2 together as markers for pericytes^20,21^. We examined markers of pericytes and endothelial cells and found that the level of marker co-expression (α-SMA and CD31) was significantly reduced in the ASDH and ASDH-DC groups (Fig. 3a). Western blot (WB) analysis also showed that the expression levels of the pericyte markers PDGFRβ, α-SMA, and NG2 were significantly lower in the ASDH group than in the sham group (p < 0.01) (Fig. 3b). Further, they remained significantly lower than in the sham group even after DC treatment (p < 0.01), suggesting that intracranial hypertension resulted in the loss of vascular wall structures by degeneration. Therefore, the extreme compression of cerebral vessels by intracranial hypertension damaged the vascular walls, capillary dysfunction remained unimproved after DC.

The expression of the microglia marker Iba-1 was determined using the Immunohistochemistry paraffin (IHC-P) assay at 12 h after the model was established. The results showed that the microglial activation was regional specificity, and the staining of Iba-1 was more obvious the closer to the hematoma side. So we selected cerebral cortex around the thickest part of the hematoma for comparison (Fig. 3c). The rate of Iba-1 staining in the ASDH and ASDH-DC groups was significantly higher than that in the sham group (p < 0.01). Next, the levels of inflammatory factors and free radicals in brain tissues from the injured side of the rat models were examined using enzyme-linked immunosorbancy assay (ELISA) (Fig. 3d). The expression of inflammatory factors and free radicals in brain tissue was significantly lower in the sham group than in the other two groups (p < 0.01). These results suggest that intracranial hypertension activates microglia, thereby mediating a cascade of neuroinflammation and oxidative stress and that this uncontrolled tendency cannot be interrupted or ameliorated despite timely relief of increased ICP.

### Cascade amplification of inflammatory response and oxidative–nitrative stress

**Figure 4:**
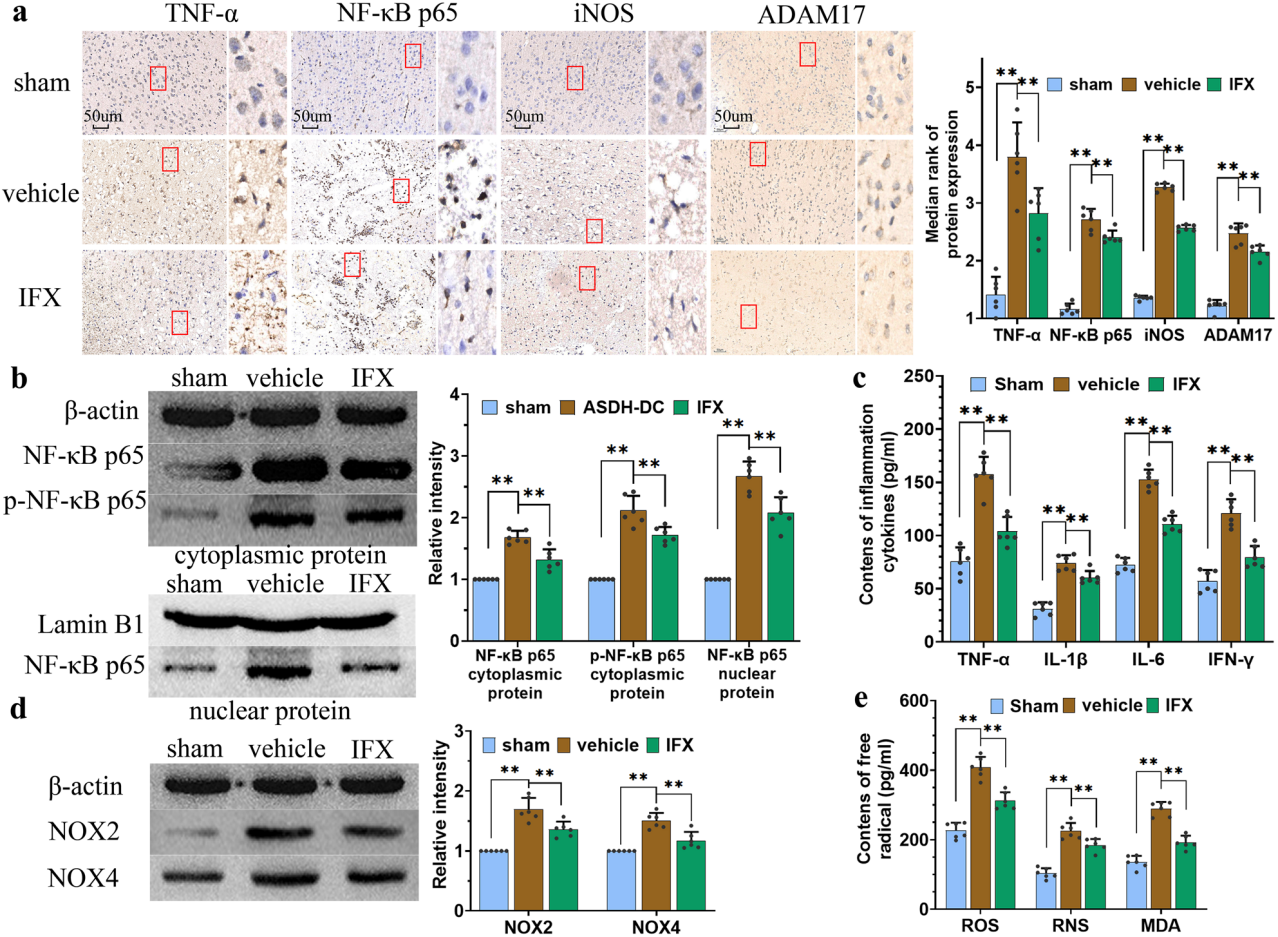
IFX inhibited the cascade amplification of inflammatory response and oxidative–nitrative stress. (**a**) Representative IHC-P images showed that noxious factors increased the expression of TNF-α, NF-κB p65, iNOS, and ADAM17 in brain tissue from the injured side, and IFX significantly downregulated the expression of these markers. The samples were taken from the same areas as Iba-1 staining (Same Fig. 3c). Red box was the magnified image. Scale bar = 50 μm. (**b**) WB analysis showed that noxious factors upregulated the expression and phosphorylation of NF-κB p65 and enhanced the nuclear translocation of NF-κB p65 from the cytoplasm, which was significantly reduced by IFX. (**c**) ELISA results showed that inhibition of TNF-α could significantly decrease ASDH-induced enhancement of inflammatory factors (TNF-α, IL-1β, IL-6, and IFN-γ). (**d**,**e**) Noxious factors led to the activation and upregulation of NOX, a major marker of excessive oxidative stress, in the rat brain. IFX decreased the expression of these factors while induced the downregulation of ROS, RNS, and MDA. Sham: the sham + DC + saline group; vehicle: the ASDH + DC + saline group; IFX: the ASDH + DC + IFX group. Values are expressed as the mean ± standard deviation (n = 6 per group), *p < 0.05, **p < 0.01.

To understand whether the intracranial hypertension can activate the TNF-α/NF-κB/iNOS signaling pathway, we evaluated key markers of this pathway using IHC-P. The results showed that the staining of key markers was more obvious the closer to the hematoma side. We also selected cerebral cortex around the thickest part of the hematoma for comparison (Fig. 4a). And the rates of TNF-α, NF-κB p65, and iNOS staining in the brain in the ASDH + DC group (vehicle group) was significantly higher than that in the sham group (p < 0.01). Additionally, the associated proteins staining was significantly decreased after infliximab (IFX) intervention (ASDH + DC + IFX drug treatment group) (p < 0.01). Moreover, the rate of ADAM17 staining was significantly different between the vehicle group and IFX group. Given that activation of the NF-κB/iNOS pathway requires nuclear translocation and phosphorylation of a large amount of NF-κB p65 to initiate the synthesis of relevant inflammatory factors, we determined its expression through WB (Fig. 4b). The results showed that NF-κB p65 was overexpressed and phosphorylated in the vehicle group and that IFX inhibited the overexpression and nuclear translocation of NF-κB p65. Meanwhile, the expression levels of inflammatory factors mitigated by IFX were also significantly decreased with the inhibition of TNF-α/NF-κB/iNOS signaling activity (Fig. 4c).

RNS, ROS, and MDA induce lipid peroxidation and protein degradation and are major markers of excessive oxidative–nitrative stress. In the present study, we detected overproduction of ROS, RNS, and MDA in the vehicle group (Fig. 4d,e). In addition, the overproduction of ROS, mainly by NOX2 and NOX4 levels were significantly higher than that the sham group (p < 0.01). In contrast, ROS, RNS, and MDA levels were significantly decreased with IFX intervention, and NOX2 and NOX4 expressions were also significantly downregulated (p < 0.01).

This indicated that intracranial hypertension induced overexpression of TNF-α and activated the NF-κB/iNOS signaling pathway in the injured brain tissues. This in turn initiated and promoted the generation of large amounts of inflammatory factors and free radicals.

### Peroxynitrite formation

**Figure 5:**
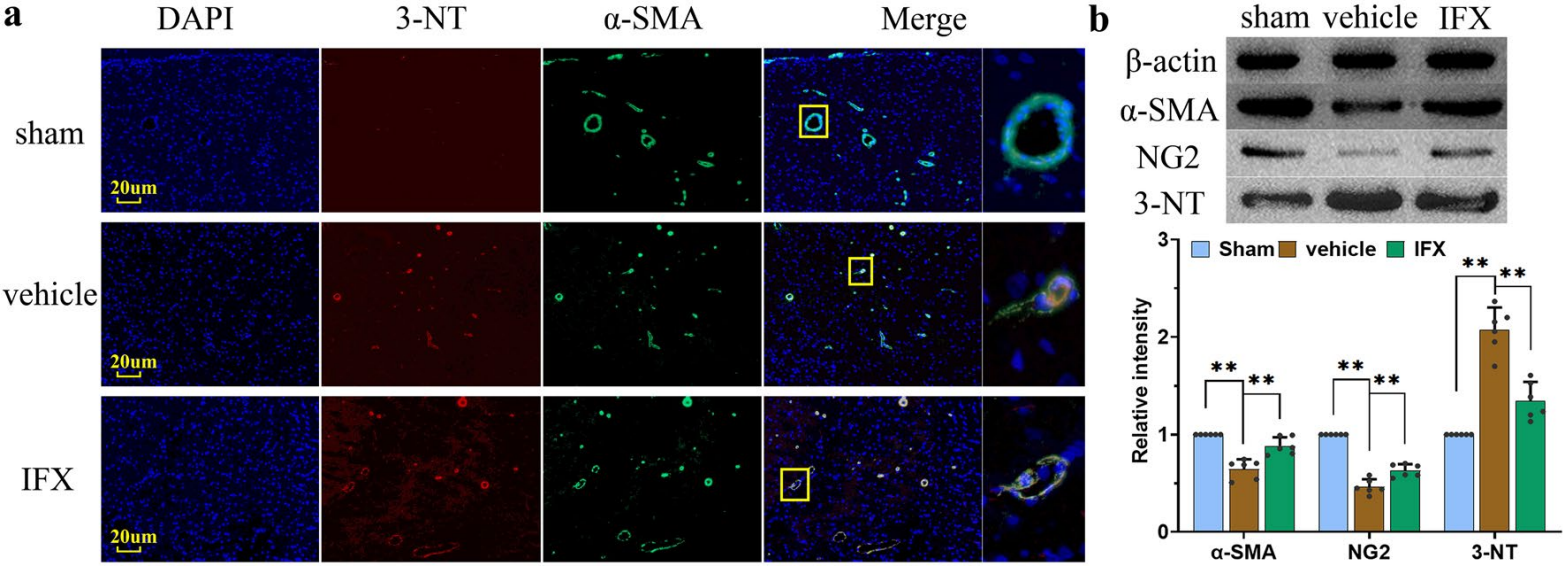
Peroxynitrite promoted degeneration and necrosis of capillary wall cells. (**a**) The markers α-SMA and 3-NT of the capillary wall cells especially pericyte were co-localized in the capillary wall near the hematoma side in the vehicle and IFX groups. The yellow boxes are enlarged colocational staining images of blood vessel wall cells (images on the far right). (**b**) WB results showed that the expression levels of α-SMA and NG2 are significantly lower in the vehicle group, whereas the expression level of 3-NT was significantly increased. After IFX intervention, the expression levels of both pericyte markers were significantly higher in the IFX group than in the vehicle group, whereas the expression levels of 3-NT were lower compared to the vehicle group. Values are expressed as the mean ± standard deviation (n = 6 per group), *p < 0.05, **p < 0.01.

Peroxynitrite reacts with tyrosine residues in proteins or free tyrosine, nitrating them into the stable metabolite 3-NT, which is believed to be a relatively specific marker of oxidative/nitrative damage mediated by peroxynitrite. Peroxynitrite is a strong oxidizing and nitrating agent that depolarizes pericytes and induces pericyte contraction and injury^12,22^. Co-localization of α-SMA and 3-NT by immunofluorescence staining is helpful to explain the destructive effect of peroxynitrite on capillary wall cells. The results showed that the staining of the peroxynitrite marker 3-NT was not observed in the capillary wall near the hematoma side of cerebral cortex in the sham group, but α-SMA and 3-NT co-localized in the capillary wall cells in the vehicle and IFX groups (Fig. 5a). Next, WB results indicated significantly lower expression of α-SMA and NG2 in the vehicle group than in the sham group, whereas the expression level of 3-NT was significantly higher (p < 0.01). The expression level of 3-NT was lower in the sham group than in the vehicle group after IFX treatment (p < 0.01). Further, the levels of α-SMA and NG2 were also significantly higher in the sham group than in the vehicle group (p < 0.01) (Fig. 5b). These results suggest that ASDH induces high levels of peroxynitrite formation in the brain, causing degeneration and necrosis of pericytes. This damage was improved after IFX intervention on pericytes during intracranial hypertension.

### BBB damage and vasogenic edema

**Figure 6:**
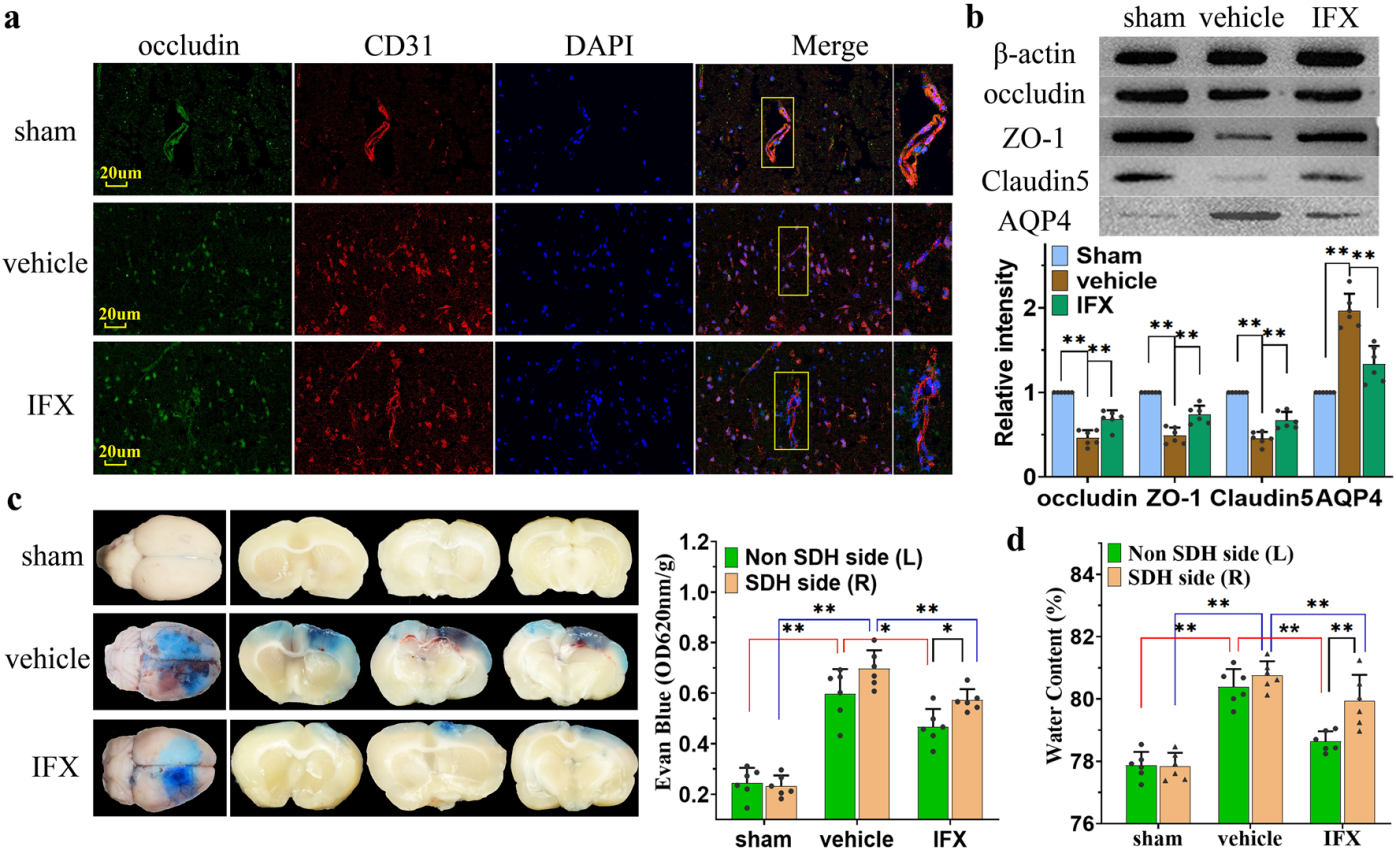
IFX ameliorated the massive destruction of the BBB in rats. (**a**) Representative fluorescence co-localized images of the TJ protein occludin and endothelial cell marker CD31 in cortical samples from the three groups of rats at 12 h after model establishment. Scale bar = 20 μm. (**b**) WB assay showed that IFX upregulated TJ protein (occludin, ZO-1, claudin5) expression and downregulates AQP4 expression. (**c**) Observation of the overall EB-stained brain tissue and coronal sections, and comparison of the proportion of EB extravasation at an absorbance of 620 nm/g. Antagonizing the TNF-α/NF-κB/iNOS pathway helps attenuate EB dye leakage, especially in brain tissue from the non-injured side. (**d**) Consistency between brain water content and EB dye leakage after brain injury. Sham: the sham + DC + saline group; vehicle: the ASDH + DC + saline group; IFX: the ASDH + DC + IFX group.Values are expressed as the mean ± standard deviation (n = 6 per group). **p < 0.01.

In traumatic brain edema, disruption of tight junction (TJ) proteins in the BBB leads to increased cerebrovascular leakage. Pericytes are embedded in the basement membranes of endothelial cells and play an important role in the expression and synthesis of TJ proteins^13,23^. Immunofluorescence staining at 12 h after model establishment showed that the morphology of blood vessel wall was marked by TJ protein occludin and endothelial cells CD31 co-localization. Compared to the sham group, the staining of blood vessel wall in the cerebral cortex near hematoma was distorted in the vehicle and IFX groups; however, the staining in the IFX group was slightly better than in the vehicle group (Fig. 6a). Next, WB results showed that TJ protein- occluding, claudin, and Zo-1 expression in the vehicle and IFX groups was significantly lower than that in the sham group; however, TJ protein expression in the IFX group was significantly better than that in the vehicle group (p < 0.01). Meanwhile, AQP4 protein expression was significantly higher in the vehicle and IFX groups than in the sham group, whereas it was lower in the IFX group than in the vehicle group (p < 0.01) (Fig. 6b).

To visualize the extent of brain edema in rats, we further investigated the effect of cranial hypertension on BBB permeability using Evans blue (EB) staining (Fig. 6c). The results showed that the amount of EB dye leakage in the bilateral brain tissues was significantly higher in the vehicle group than in the sham group. Meanwhile, the amount of EB dye leakage in the brain tissue on the injured side was significantly lower in the IFX group than in the vehicle group (p < 0.01). In addition, there was a difference in the amount of EB dye leakage in the brain tissue on the non-injured side between the IFX and vehicle groups and between the two sides within the IFX group (p < 0.05). Similarly, brain tissue water content were consistent with EB staining results in all groups (Fig. 6d). These results suggest that the intracranial hypertension leads to disruption of BBB structure, resulting in severe vasogenic edema of brain tissue.

### Disturbance of microcirculatory blood flow

**Figure 7:**
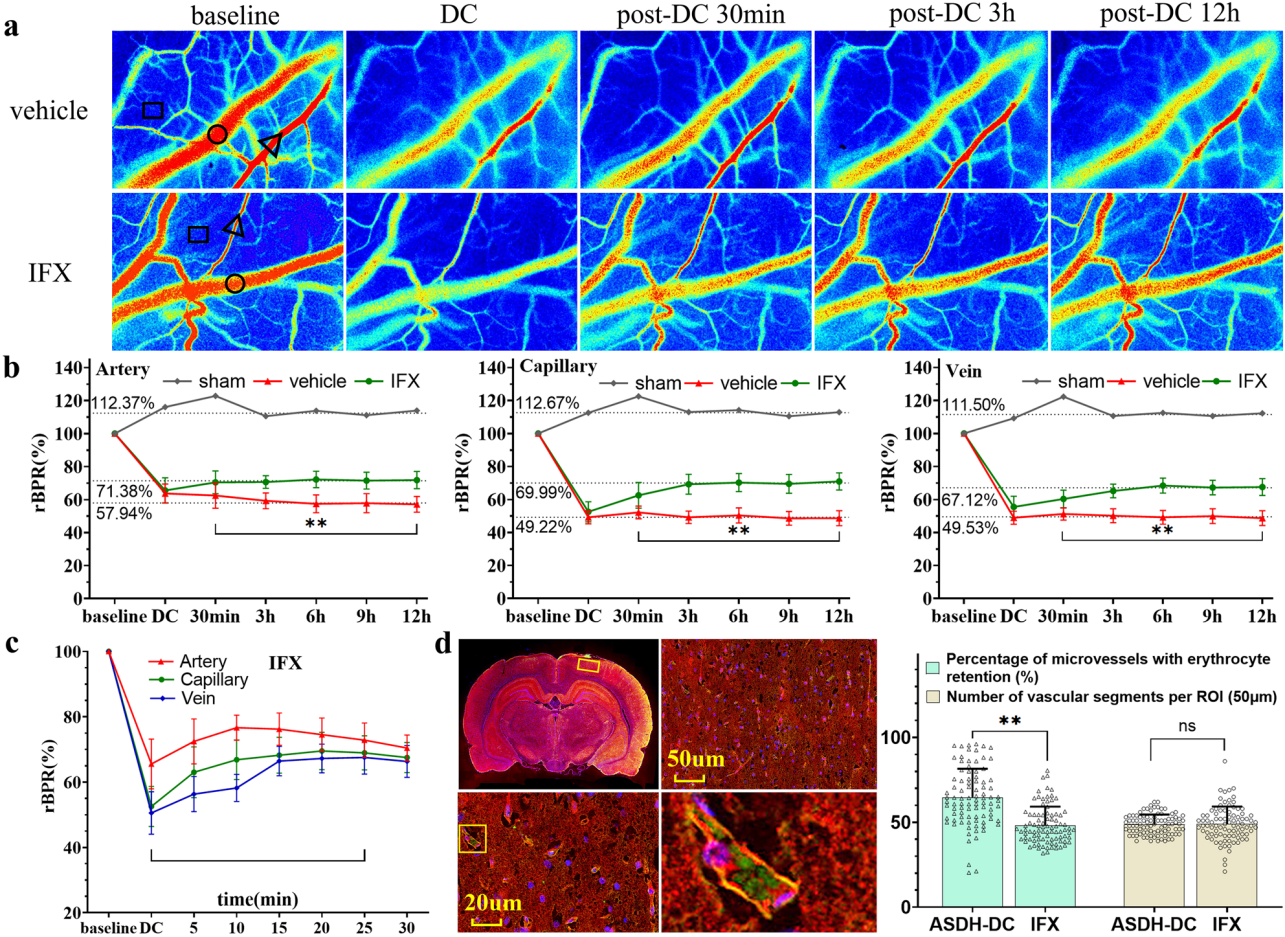
IFX attenuated microcirculatory reperfusion in the cerebral cortex on the injured side. (**a**) LSCI monitoring of the ROIs on the cerebral cortex near the hematoma side before and 12 h after DC. Triangles, circles, and squares indicate CBF monitoring sites in arterial, venous, and capillary regions, respectively. (**b**) Dynamic curve of the changes in rBPR in different vessels of the cerebral cortex on the hematoma side in the three groups of rats. (**c**) Dynamic curve of the changes in different vessels at 30 min after DC in the IFX group. (**d**) Blood deposition was better in the IFX group than in the ASDH-DC group, and there were no difference in total vessel counts. vehicle: the ASDH + DC + saline group; IFX: the ASDH + DC + IFX group. Values are expressed as the mean ± standard deviation (n = 6 per group). **p < 0.01.

To understand the effect of pericyte injury on circulatory perfusion in the cerebral cortex, we observed CBF of different vessel types on the injured side for 12 h after DC (Fig. 7a). The results showed that in the sham group, the rBPR of all cortical vessels rapidly increased and then gradually decreased over time, but they remained higher than baseline values. In addition, the changes in arteries, veins, and capillaries were nearly synchronous. The vehicle and IFX groups exhibited a significant decrease in rBPR in all vessel segments after model establishment, and CBF did not increase significantly after DC in any vessel segments, remaining far from baseline. The rBPR of each vascular segment in the IFX group was not significantly different compared to that in the vehicle group in 30 min, but it was slightly elevated at 3–12 h, where in the rBPR of all vascular segments were significantly higher in the IFX group than in the vehicle group (p < 0.01) (Fig. 7b).

We also analyzed the rBPR of each vascular segment in the IFX group within 30 min after DC. The rBPR of arterial segments was rapidly increased after DC and then gradually decreased during brain swelling. The rBPR of capillary and venous segments exhibited gradual recovery along with arterial segments (Fig. 7c). Based on recovery curves, recovery in capillary or venous segments was not significant delayed compared to that in the ASDH-DC group (Fig. 1F), but it remained below baseline levels. For red blood cell deposition in capillaries in the IFX and ASDH-DC groups (Fig. 7d), the results indicated a significant reduction in red blood cell deposition in the IFX group (p < 0.01) but no significant difference in the number of microvessels within the ROI was found between the two groups. This indicates that IFX antagonizes pericyte damage, attenuates capillary dysfunction, and reduces blood cell deposition, thereby increasing the rate of capillary and venous responses to DC.

## Discussion

**Figure 8:**
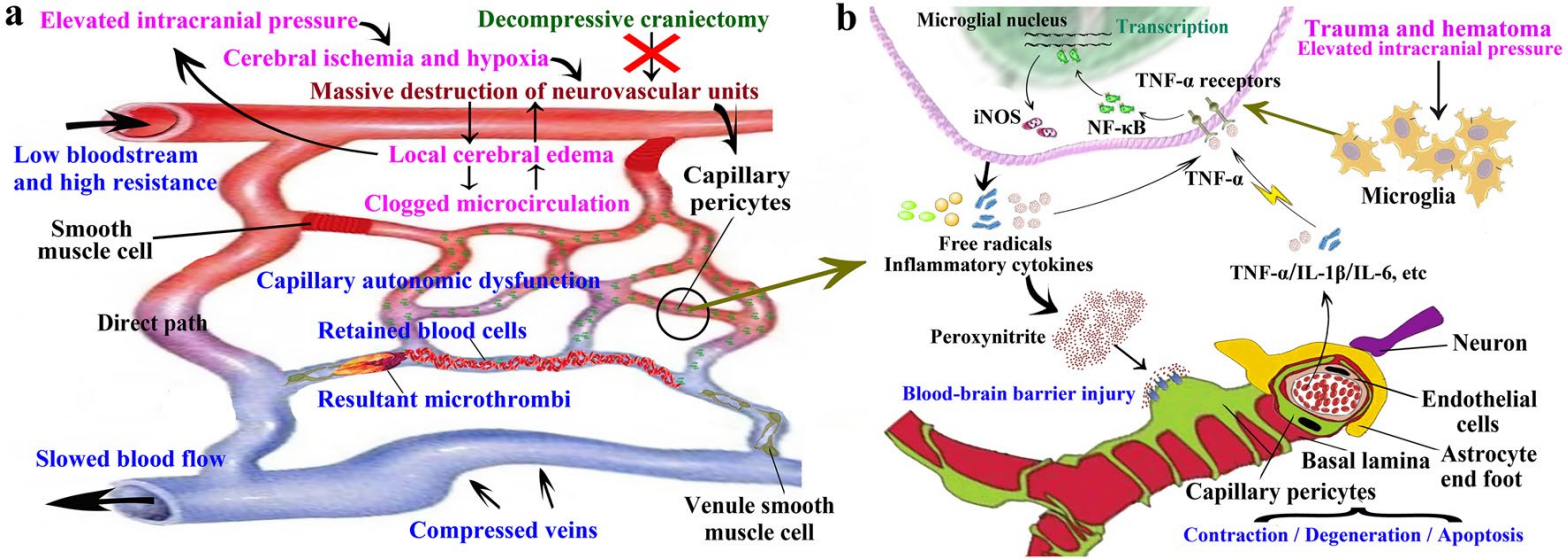
Schematic illustration of the mechanisms. (**a**) Microglial activation amplifies the cascade of inflammatory and oxidative–nitrative stress responses through the TNF-α/NF-κB/iNOS axis, generating large amounts of peroxynitrite, which induces pericyte injury and capillary dysfunction, resulting in blood cell deposition and microthrombosis. This delays the recovery of microcirculatory blood flow and ultimately exacerbates DBS or intraoperative brain herniation. (**b**) The sudden increase in ICP reduces arterial blood flow, produces ischemia and hypoxia in the brain, and destroys a large number of NVUs. Local cerebral microcirculatory dysfunction causes severe disruption of the BBB, further exacerbating the increase in ICP and creating a vicious cycle in which DC cannot stop the course of secondary brain injury.

This study evaluates the cerebrovascular and perivascular alterations under acute intracranial hypertension in TBI, further explaining the mechanism of DBS or intraoperative brain herniation during DC. Traumatic ASDH is highly likely to cause a sudden increase in ICP, leading to microglia activation, cascade amplification of the inflammatory factor and oxidative–nitrative stress response via the TNF-α/NF-κB/iNOS axis. A significant amount of peroxynitrite formation causes severe damage to capillary wall cells especially pericytes and the BBB, impairing the autonomic regulation of capillaries (Fig. 8a). This leads to deposition of red blood cells and can even result in microthrombosis in the capillary lumen. At this point, the veins are compressed, pulled, twisted, and injured, resulting in differences in the rates of perfusion across the cortical arterioles, capillaries, and veins on the injured side at the time of DC, which contributes to DBS of the injured brain tissue and intraoperative brain herniation (Fig. 8b). In addition, this study confirmed that IFX treatment resulted in downregulation of inflammatory and oxidative–nitrative stress-related factors, attenuation of pericyte and BBB injury, and relative reduction of capillary hemostasis, shifting and approaching of blood recovery curves between arteries with capillary and venous, thereby reducing the extent of DBS or intraoperative brain herniation during DC.

Neuropathological studies have shown that the mortality rate of traumatic ASDH is associated with ischemic damage to the cerebral hemispheres under the hematoma^4,24–26^. The key contributing factor to this phenomenon is the sudden increase in ICP caused by the hematoma, which severely limits cerebral arterial perfusion. This was evident in the early stages following ASDH hematoma injection in the rat model^5,7,27^. Arterial and capillary blood flow can recover partially as ICP decreases but not venous blood flow owing to the lack of internal elastic lamina and the compressive effects of the hematoma, which results in blood deposition in the brain. Such ischemic-hypoxic changes are the initiating factor for secondary injury of brain cells. Ca^2+^ overload, toxic effects of oxygen radicals, impaired energy metabolism, excitotoxicity, cytokine release, and autonomic dysfunction result in massive destruction of the NVUs and severe cerebrovascular autonomic dysfunction^28–30^. DC is a standard intervention for the treatment of traumatic ASDH^31,32^. Blood flow in arterial segments is rapidly improved after DC. However, capillary and venous segments have a markedly delayed recovery of blood flow. The blood flow is even more severely delayed compared to the early stages of hematoma formation. Similar results have been found in other studies^7,27,33^. This heterogeneous response in the cerebral vasculature results in rapid swelling due to hyperperfusion of cerebral tissue and is the principal cause of malignant intraoperative brain herniation in DC.

In the capillary and venous segments, the capillary wall cells, especially the pericapillary cells, are surrounded by the basal lamina and play a major role in the regulation of blood flow in the microcirculation through neurovascular coupling^13,17,34,35^. In stroke disease models, intense contraction of pericytes in the ischemic core as well as in the penumbra is observed in the early stage^11,36,37^. This resulted in narrowing of the capillary lumen and restricted passage of blood cells through the lumen, increasing the oxygen exchange capacity of the local region. However, sustained intense contraction resulted in the eventual death of the pericytes, causing loss of blood flow regulation in the capillary segment as well as blood cell deposition. Our results indicate that under sudden intracranial hypertension, the response of pericytes is similar to that in stroke, with significant degeneration, necrosis, and loss of pericytes with the onset of cerebral ischemia and hypoxia. Further, a large number of blood cells are deposited in the capillary segment and blood cells are blocked, with microthrombosis even occurring in the microvessels. These were not improved by DC and severely affected cerebral reperfusion, ultimately resulting in delayed blood flow in the capillary segment.

Pericytes die from injury induced by the inflammatory response and oxidative–nitrative stress^11,12,38^. Microglia are the immune sensors of the central nervous system, which causes neurological damage through the secretion of pro-inflammatory factors and ROS/RNS^8,14,39,40^. We found that inflammation-associated microglia were activated and various inflammatory factors, including TNF-α, IL-1β, IL-6, and IFN-γ, as well as ROS/RNS, were released. This was not significantly ameliorated after DC intervention, suggesting that sustained extreme intracranial hypertension amplifies the inflammatory response and oxidative–nitrative stress. NF-κB efficiently induces the expression of inflammatory cytokines, chemokines, adhesion factors, and inflammatory enzymes, resulting in cascade amplification of inflammatory responses and oxidative–nitrative stress^41–43^. Similarly, we found that the NF-κB/iNOS pathway was also activated along with increased NF-κB phosphorylation and nuclear translocation. The use of the TNF-α-conjugated monoclonal antibody IFX downregulated the expression of the NF-κB/iNOS pathway, attenuating inflammatory cytokine release and degradation of related proteins in the brain.

Both RNS and ROS molecules can interact with each other to form peroxynitrite. Peroxynitrite is another toxic substance that destroys intracellular macromolecules such as proteins, lipids, and nucleic acids, thereby causing mitochondrial dysfunction and elevated intracellular calcium ions^11,38,44–46^. We found that IFX reduces peroxynitrite formation by decreasing oxidative–nitrative stress, resulting in reduced pericyte degeneration and injury.

Pericytes closely interact with the vascular endothelium and are associated with transendothelial fluid flow and paracellular transport across the BBB. Thus, any injury or dysfunction of the pericytes results in severe vasogenic edema and increased intracellular water content^13,47–49^. The cerebral microcirculation is the largest vascular network in the brain, and the reactions in different types of vessels when subjected to extravascular pressure depends on the density and distribution of smooth muscle. Extravascular pressure directly affects the function of the vasculature of the cerebral microcirculation^50,51^. That is, brain tissue that is highly compressed by ASDH experiences extremely high ICP before DC, inevitably increasing the extravascular pressure on cerebral blood vessels and weakening their blood supply capacity. This in turn causes ischemia and hypoxia of brain cells and promotes malignant cerebral edema, further increasing ICP, thus forming a vicious cycle. In the present study, inhibition of the TNF-α/NF-κB/iNOS axis significantly reduced the loss of TJ proteins, and upregulated the expression of AQP4. These effects helped protect the structural and functional integrity of the BBB, thereby attenuating the synergistic effects of cerebral microcirculatory dysfunction and malignant cerebral edema.

In conclusion, intracranial hypertension induced by traumatic ASDH causes an inflammatory factor storm and oxidative–nitrative stress that result in capillary wall cells degeneration and injury, especially pericyte, deposition of a large number of blood cells in the lumen of the microvessels, and disruption of the BBB. These processes result in delayed recovery of capillary and venous flow, which in turn plays an essential role in the development of diffuse cerebral swelling and malignant brain herniation after DC. In contrast, inhibition of inflammatory and oxidative–nitrative stress responses ameliorates capillary wall cells injury and cerebral microcirculatory dysfunction, thereby reducing the extent of DBS and malignant cerebral edema.

### Limitations of the study

First, we did not identify direct pathways and arteriovenous short-circuit structures in the microcirculation that enable direct blood flow into the veins (Fig. 8B). These may help mitigate delayed synchronization of microcirculation^52^. Further, we have also neglected the fact that other cells in the NVUs, especially astrocytes and endothelial cells, may have a closer spatial relationship with pericytes^49,53,54^. Second, the JAK/STAT signaling axis to recruitment and activation of inflammatory factors and nuclear factor E2-related factor 2/antioxidant factor element signaling pathway to endogenous resistance to oxidative stress have not been studied with respect^55,56^. The associated mechanisms do not act independently but instead are intricately linked and interact with each other. It is unclear whether or how pericytes change phenotypes between mechanisms; the current study focused only on the role of the NF- κB/iNOS axis. Third, the effects of an effective and safe dose of IFX and the therapeutic time window on its efficacy in the rat model have not been established to date, which brings into question the clinical translation of the drug. Our IFX intervention was provided at an earlier timeframe, and the dose was higher. Further research is needed on the molecular mechanisms of DBS and malignant brain herniation after DC.

## Materials and methods

### Animals and grouping

Healthy adult rats (Sprague Dawley, Slack Laboratory Animal Co., LTD, Shanghai, China) weighing approximately 270–320 g were housed in cages under pathogen-free conditions at the Laboratory Animal Center of the 900 Hospital. All rats were housed and cared for by specialized personnel under the same rearing conditions. All animals were randomized for group allocation and surgical procedures, and all surgical procedures were standardized. The operators responsible for the data analysis were blinded and unaware of group allocation throughout. The animal experiment was approved by the Experimental Animal Welfare Ethics Committee of the 900th Hospital (Number: 2020-050) and conducted in accordance with ARRIVE guidelines (PLoS Bio 8(6), e1000412, 2010)^57^. All methods were performed in accordance with the relevant guidelines and regulations.

In the first part of the study, adult rats were randomly divided into sham, ASDH, and ASDH + DC groups (n = 24 per group). Eighteen of each group were randomly monitored for physiological data, and paraffin sections of the brain were produced after monitoring (n = 6 per subgroup). The remaining six of each group was used for WB analysis and ELISA. In the second part of the study, adult rats were randomly divided into sham, ASDH + DC group (vehicle group), and ASDH + DC + infliximab (IFX) drug treatment group (IFX group) (n = 18 per group). Each group was randomly divided equally into four subgroups. One subgroup (n = 6) was used for WB analysis, ELISA, and brain water content assessment. One subgroup (n = 6) was used for physiological data monitoring, and paraffin sections of the brain were produced after monitoring. Last subgroup (n = 6) was used for the EB extravasation assay.

### Model development and drug administration

All rats were anesthetized with 3% isoflurane gas, immobilized on a stereotactic frame, and subjected to continuous mechanical ventilation with a gas mixture containing 78% nitrogen gas, 21% oxygen gas, and 1% isoflurane using a 16G polyurethane tracheal intubation catheter (Tracheal intubation suit RE20, Intelligent animal ventilator R419, RWD Life Science Co., Shenzhen, China). All rats were drilled with a high-speed drill (ALC-CED8, Alcott Biological Co., Shanghai, China) with a 1.3-mm hole (one on each side) at 1.5 mm anterior to the coronal suture and at 2 mm lateral to the sagittal suture. To ensure the survival of the rats with severe TBI, anesthesia and mechanical ventilation were continued until the rats were killed.

The ASDH model was created based on previous methods described by Xian and Zhang^27,58^. Briefly, the dura mater was cut at the right craniotomy hole, and an 8-G gavage needle with an external diameter of 1.4 mm was placed close to the craniotomy hole and the surrounding seal was maintained. Subsequently, 400 µL of semi-coagulated blood was injected under the dura mater at a rate of 80 µL/min and the craniotomy hole was closed with bone wax. Hematomas were derived from autologous fresh blood drawn from the femoral vein and placed in a 37 °C fixed-temperature water bath for 3 min, during which the blood clot developed into a jelly like semi-coagulated state. The DC model was performed 4 h after the ASDH model was completed. A bone flap above the hematoma between the coronal and lambdoid sutures was deflected to the right side of the sagittal suture, with a diameter of approximately 6 × 5 mm. The dura was cut open, the hematoma was evacuated, exposing the cerebral cortex.

In the first part of the study, the rats in ASDH group were only performed by the ASDH model operation, and the rats in sham and ASDH-DC groups were performed by the ASDH model and DC model operation successively. In the second part, all rats were performed by the ASHD model and DC model operation successively. Meanwhile, the rats in sham and vehicle group were injected with normal saline (20 ug/g) 30 min after model establishment, and the IFX group rats were administered an intraperitoneal injection of IFX (diluted with normal saline to 2.5 mg/ml, 20 ug/g, cilag Ag)^38,58,59^.

### Monitoring of physiological data

An ICP probe was implanted into the left craniotomy hole and connected to a pressure monitor (Type 82-6631, NicoletOne monitor; Codman, Johnson & Johnson Inc., USA). A 21-G heparin-containing femoral artery catheter was placed in the right femoral artery and connected to a small animal vital sign monitor (ALC-MPA-MS, Alcott Biological Co., Shanghai, China) for continuous acquisition of ICP and MAP data. Cranium polishing was performed between the coronal and left lambdoid sutures to create an observation zone, with care taken to maintain the integrity of the cranium and to achieve good laser penetrability. A laser speckle contrast imaging (LSCI) system (RFLSI III, RWD Life Science Co., Shenzhen, China) was used to identify the arteries, microcapillaries, and veins by vessel color, branching, and direction of blood flow in the cerebral cortex vascular viewing window. ROI were randomly selected, and the diameters and blood flow values of each vessel segment were measured and recorded using a software tool. The dynamic blood perfusion rates of the observed vessels were quantified using the formula: $Q = {{V \times \pi \times {D^2}} /4}$, where V is equal to the blood flow velocity and D is equal to the vessel diameter. Subsequently, the dynamic Q values and baseline values were normalized to obtain the rBPR values^27,58,60^.

### Preparation of paraffin-embedded sections

Twelve hours after model establishment, all rats were deeply anesthetized with an overdose of 3% isoflurane gas, placed in the supine position, and immobilized on the operating table. The abdominal cavity was opened into the thorax to completely expose the heart. The rats were exsanguinated by implanting a cannulated needle in the left ventricle and cutting the right auricle. Phosphate-buffered saline was rapidly perfused through the cannula until the liver turned white and clear and transparent fluid flowed out of the right auricle. We then switched to a continuous drip of 4% paraformaldehyde until the rats were stiffened. The brain was removed and fixed by immersion in 4% paraformaldehyde solution for 48 h. Next, the brain tissue was embedded in paraffin, oriented in the coronal plane, and sliced into 4-μm sections for further immunohistochemical and immunofluorescence staining.

### Immunohistochemistry paraffin (IHC-P)

The paraffin sections were processed by deparaffinization, dehydration, antigen repair, and blocking. Next, primary antibodies, including anti-Iba-1 (1:500, ab178847, Abcam, UK), TNF-α (1:1000, ab307164, Abcam), NF-κB p65 (1:200, ab239882, Abcam), iNOS (1:250, ab178945, Abcam), and TNF-α invertase ADAM17 (1:200, ab39162, Abcam) were added dropwise, followed by the addition of secondary antibodies (1:500, ab197740, Abcam). The slides were then placed in an incubator, developed for color, and mounted. Three sections were selected for each rat, and five ROIs around the injured lesions in each section were randomly selected for light microscopy observations at a scale of 50 μm. The signal intensity was evaluated using the ImageJ software as follows: 0, < 5% positive cells; 1, 5–20% positive cells; 2, 20–50% positive cells; 3, 50–75% positive cells; and 4, > 75% positive cells.

### Immunofluorescent staining (IF)

The paraffin sections were processed by deparaffinization, dehydration, antigen repair, and blocking. The slides were incubated with antibodies against ZO-1 (1:100, ab221547, Abcam), FITC Anti-Hemoglobin antibody (1:100, ab19361, Abcam), α-SMA (1:500, ab124964, Abcam), PECAM-1/CD31 (1:200, ab281583, Abcam), occludin (1:1000, ab216327, Abcam), 3-NT (2 µg/ml, ab110282, Abcam), and stained. Nuclei were stained with 4′,6-diamidino-2-phenylindole, and the slides were rinsed, shaken, dried, and mounted. Three sections were selected for each rat, and five ROIs around the injured lesions in each section were imaged under a fluorescence microscope (TCS SP8, Leica Microsystems, Germany).

### Enzyme-linked immunosorbent assay (ELISA)

After euthanasia, 10 mg of the brain tissue near the injury site was collected and frozen. Blood on the surface of the samples was removed, and the samples were homogenized and centrifuged at 4 °C for 5 min. The supernatant was collected by aspiration, and TNF-α, IL-1β, IL-6, interferon-gamma (IFN-γ), ROS, RNS, and MDA levels were determined using ELISA kits (Jingmei Biotechnology, China) per the manufacturer’s instructions.

### Western blot analysis (WB)

The same amount of brain tissue was collected from the injured cerebral hemisphere, weighed, completely lysed in RIPA lysis buffer containing a mixture of protease and phosphatase inhibitors, and centrifuged. The supernatant was collected and cooled until use. After high-speed centrifugation following the instructions of the Nuclear and Cytoplasmic Protein Extraction Kit (KGP1100, KeyGEN, China), the supernatant (cytoplasmic fraction) and the precipitate (nuclear fraction) were isolated. The protein concentration of the extracted samples was determined using a BCA kit, according to the manufacturer’s instructions. After protein denaturation, the target proteins were separated by sodium dodecyl sulfate–polyacrylamide gel electrophoresis, transferred to a polyvinylidene fluoride membrane (Immobilon-P, Millipore, Bedford, MA, USA), and blocked. Next, the bands were incubated with primary antibodies against α-SMA ( 1:500, 19245T, CST), PDGFR-β (1:500, 3169T, CST), NG2 (1:1000, ab275024, Abcam), NF-κB p65 (1:1000, ab239882, Abcam), p-NF-κB p65 (1:1000, ab86299, Abcam), NOX 2 (1:1000, ab310337, Abcam), NOX 4 (1:1000, ab154244, Abcam), 3-NT (1:1000, ab61392, Abcam), ZO-1 (1:500, ab276131, Abcam), occludin (1:1000, ab216327, Abcam), claudin-5 (1:1000, ab131259, Abcam), or aquaporin (AQP) 4 (1:1000, ab259318, Abcam). Grayscale images of the bands were obtained using an enhanced chemiluminescence (ECL) western blotting detection system. The grayscale values of the bands were analyzed using ImageJ and normalized to β-actin (1:2000, ab8226, Abcam) or Lamin B1 (1:2,000, ab16048, Abcam) bands. Finally, the relative amounts of target proteins were determined.

### Evans blue staining (EB)

Rats were injected with Evans blue dye (2%, 5 ml/kg, Sigma-Aldrich, USA) via the femoral vein prior to euthanasia. After 2 h, cardiac perfusion was performed with heparinized saline, the rats were decapitated, and their brains were removed. Specimens were cut into six coronal sections, starting from the occipital lobe forward, and observed for EB dye leakage. Subsequently, the brain tissue on both sides was evenly isolated, weighed, cut into small pieces, incubated in dimethylformamide solution (1 mL/100 g) for 24 h at 60 °C for 24 h, and centrifuged at 1000 r/min for 5 min. The absorbance at 620 nm was measured using a spectrophotometer, and the EB content of each brain tissue slice was quantitatively calculated after plotting the standard curve.

### Assessment of cerebral edema

Brain tissue specimens were evenly isolated from the median sagittal plane, cleaned of surface blood using filter paper, wrapped with pre-weighed aluminum foil, and weighed on an electronic analytical balance (subtracting the weight of the aluminum foil) to obtain the wet weight. Next, the brain tissue was dried in an oven at 90 °C for 72 h until there was no further weight loss and then weighed again to obtain the dry weight. Brain tissue water content = (wet weight-dry weight)/wet weight × 100%.

### Statistical analysis

All data are expressed as the mean ± standard deviation. Images were created using the GraphPad Prism software (version 8.0). The Shapiro–Wilk test was used to determine the normality of the data distribution. Comparisons between two groups were performed using the independent samples t-test, and among the three groups using one-way analysis of variance. Pairwise comparisons between the groups were performed using the Bonferroni method. All statistical analyses were performed using the SPSS software (Version 26.0, SPSS Inc. Chicago, IL, USA). Differences with P (two tail) < 0.05 were considered statistically significant at p < 0.05.

### Supplementary Information

Supplementary Information.

## Data Availability

The raw data supporting the conclusions of this article will be made available by the authors, without undue reservation. Part of the data is provided in Supplementary Info File. Please contact the author (zhangshangming2019@163.com and wshsen1965@126.com) if anyone need more data.

## Abbreviations

TBI: Traumatic brain injury
ASDH: Acute subdural hematoma
ICP: Intracranial pressure
DBS: Diffuse brain swelling
DC: Decompressive craniectomy
NVU: Neurovascular unit
BBB: Blood–brain barrier
TNF-α: Tumor necrosis factor α
NF-κB: Nuclear factor-κB
iNOS: Inducible nitric oxide synthase
IFX: Infliximab
MAP: Mean arterial pressure
ABG: Arterial blood gases
CBF: Cerebral blood flow
rBPR: Relative blood perfusion rate
IF: Immunofluorescent
ZO-1: Zonula occluden 1
ROIs: Regions of interest
PDGFRβ: Platelet derived growth factor receptor β
α-SMA: α-Smooth muscle actin
NG2: Neuron-glial 2
CD31/PECAM-1: Platelet endothelial cell adhesion molecule-1
IHC-P: Immunohistochemistry paraffin
WB: Western blot analysis
IF: Immunofluorescent staining
IFN-γ: Interferon-gamma
IL: Interleukin
Iba-1: Ionized calcium-binding adapter molecule 1
ELISA: Enzyme-linked immunosorbancy assay
ADAM17: A disintegrin and metalloproteinase 17
ROS: Reactive oxygen species
RNS: Reactive nitrogen species
MDA: Malondialdehyde
NOX: Nicotinamide adenine dinucleotide phosphate (NADPH) oxidase
3-NT: 3-Nitrotyrosine
TJ: Tight junction
AQP: Aquaporin
EB: Evans blue
JAK/STAT: Janus kinase/signal transducer and activator of transcription
LSCI: Laser speckle contrast imaging
RIPA: Radioimmunoprecipitation assay
LPS: Lipopolysaccharide
CNS: Central nervous system
DAPI: 4′,6-Diamidino-2-phenylindole
ANOVA: One-way analysis of variance
