# Supplementary material for: Assessment of cerebrovascular alterations induced by inflammatory response and oxidative–nitrative stress after traumatic intracranial hypertension and a potential mitigation strategy
Source: Sci Rep. 2024 Jun 24;14:14535. doi: 10.1038/s41598-024-64940-6 (PMC11196732; doi:10.1038/s41598-024-64940-6)
Supplement: Supplementary file 1 — Supplementary Information. [file 41598_2024_64940_MOESM1_ESM.pdf]

Supplementary Document

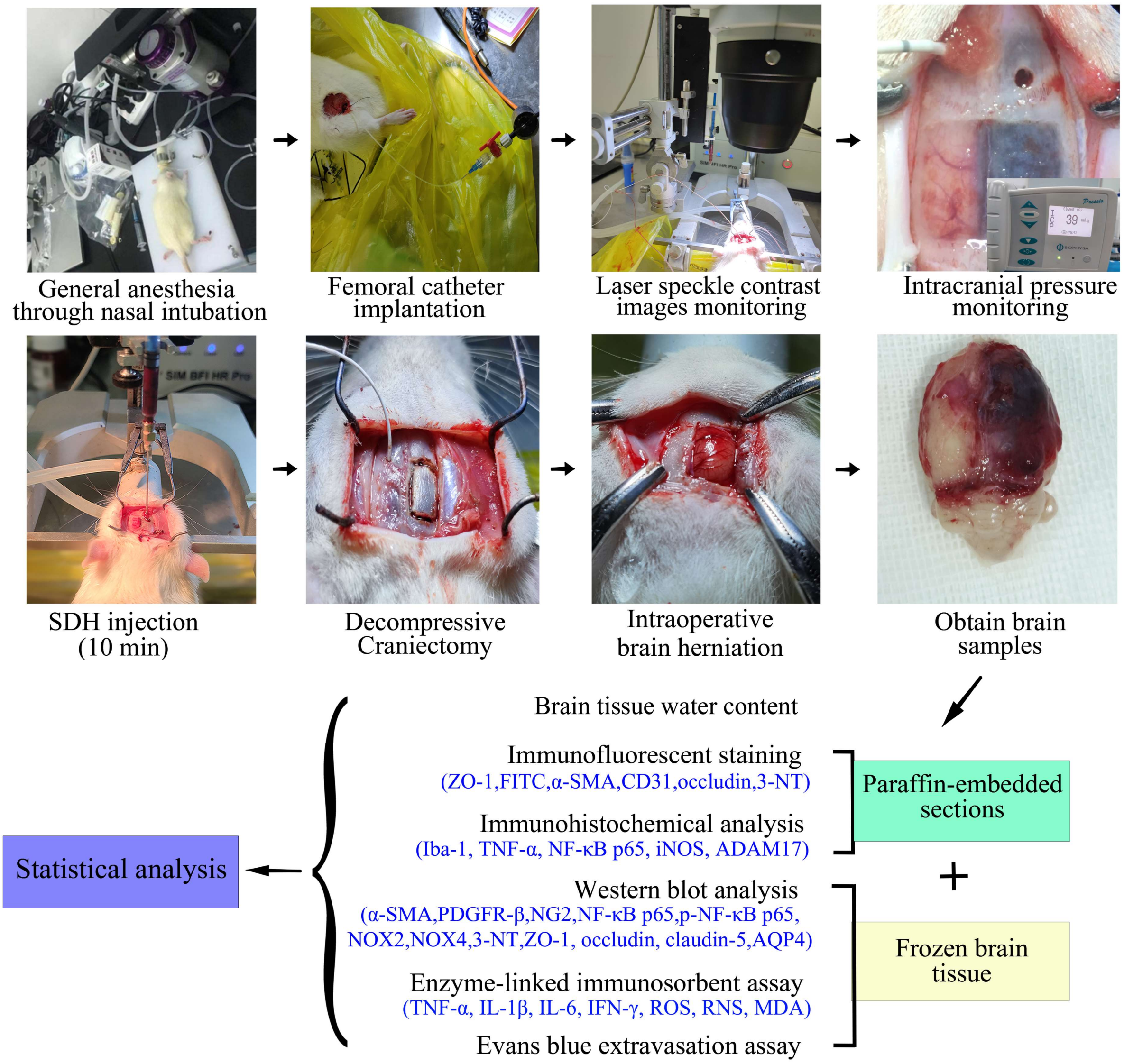

Figure 1 Experimental protocol of the rat model.

Western-bolt reports (PART ONE)

1. Experimental instruments and reagents

	**ITEM NO**	**Manufacturer or brand**
Color predye protein molecular weight	P0069	Posterity And Perplexity
BCA Protein Concentration Assay Kit (50T)	PC0020-500	Beijing Solaibao Technology Co., LTD
ECL 发光试剂	34080	Thermo
5×蛋白上样缓冲液	P1041	Beijing Solaibao Technology Co., LTD
PVDF 膜（0.22um）	ISEQ00010	Millipore
β-actin antibody	ab8226	abcam
Anti-alpha smooth muscle Actin antibody	Ab124964	abcam
Anti-NG2 antibody	Ab275024	abcam
PDGFRβ antibody	MA00096	Boster

2. Experimental steps

Sample preparation

The tissue samples were added with protein lysate containing PMSF, and the samples were placed on ice for 20 min, centrifuged at 12 000 rpm at 4℃ for 20 min, and 400 μL of the superalbumin solution was taken into an EP tube.

Sample protein determination and denaturation

The total protein concentration was detected by BCA protein assay. Take 5 μL sample and dilute it 20 times with three steaming water, mix it well, take 20 μL each of the diluted protein sample and the standard product, and take 160 μL of the BCA working liquid, add it into the 96-well protein determination plate (for 2 repetitions), and place it in the 37℃ incubator for 30 min. Then, the absorbance of each sample is detected by enzymoleter, and the protein concentration of each sample is calculated. After the protein sample was diluted with RIPA lysate to the same concentration, the sample was 100 μL in volume, and the loading buffer was 20μL. Then the sample was fully mixed, denatured in water bath at 98℃ for 5 min, and stored at -80℃ after ice cooling.

Sds-polyacrylamide gel electrophoresis

Protein molecular weight of different configurations of different concentrations of separation glue 10%,

the concentrator is 5%. After the SDS-page glue was completely solidified, the comb was pulled out, the gel was put into the electrophoresis tank, and the electrophoresis solution was filled. After sampling, the voltage was stabilized at 90 V for 40 min and the voltage was adjusted to 120 V after the protein Marker was dispersed. After electrophoresis, the target strip was cut.

Transfer printing

Protein transfer: Prepare 8.5 × 6.5cm PVDF film and 8 × 6cm filter paper. After marking, soak in the transfer buffer for 15-30 min. Then fix the filter paper, separation glue, nitrate cellulose film and filter paper in the splint and remove bubbles in the order from bottom to top. Fill the transfer tank with transfer liquid and transfer to the cold storage. Transfer conditions: 4°C, 100 V, 90 min.

close

The transferred PVDF membrane protein was placed face-up in 5% Albumin from bovine serum (BSA), placed in a 40 rpm shaker and closed at room temperature for 2 hours.

Antibody incubation

After closure, PVDF membrane was placed into antibody hybridization box containing primary antibody (β-actin 1:1000, PDGFRβ 1:1000, α-SMA 1:10000, NG2 1:1000) and incubated overnight at 4°C in a shaking table. On the second day, the incubated PVDF membrane was washed with TBST for 3 times, 10 min each time. After cleaning, PVDF membrane was placed into a rabbit two-antibody hybridization box diluted with l:5000, and incubated at room temperature on a horizontal shaking table for 2 h; If the room temperature is high, the time can also be shortened appropriately. After the end, also wash with TBST 3 times, 10 min each time.

Antibody conjugated HRP detection

The PVDF film was placed on the transparent film, and the Thermo ECL kit's liquid A and liquid B were mixed and added to the surface of the film. After standing in the dark for 3 minutes, the luminescent liquid on the film was removed and placed on the transparent film. A gel imaging system was used for luminescence detection and strip images were collected.

Strip analysis

Image J analyzed the band gray values to determine the relative content of target protein expression in the sample.

3. Experimental results

3.1 PDGFRβ

Sample1	Sample2	Sample3

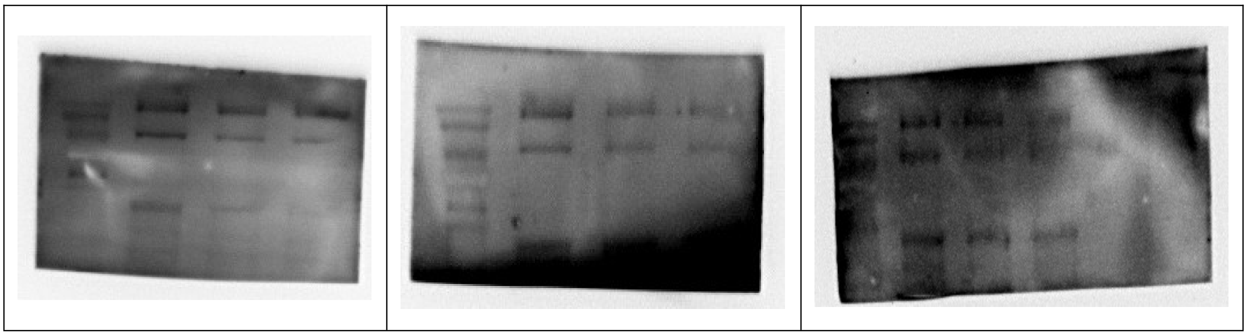

PDGFRβ	Sham	1	19143.602	1	46475.551	1	49677.016		0.819349882	2.371390446	0.993987595	1.394909308	1	1	1		1	0.609468727	
	ASDH	2	25290.451	2	17126.966	2	52861.714		1.127530674	0.484086846	1.29526446		0.808318267	0.347038222	0.928565358		0.694640616	0.306977863	0.481582565
	ASDH-DC	3	24559.966	3	2674.276	3	38039.957		0.733391467	0.074714246	0.966807042		0.525762832	0.053562082	0.693096703		0.424140539	0.331657128	

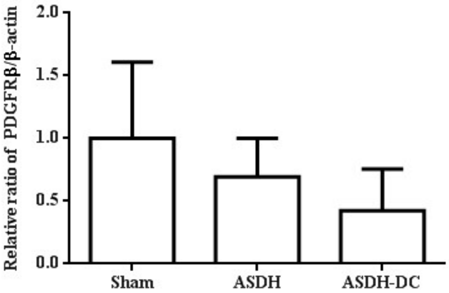

3.2 α-SMA

Sample1	Sample2	Sample3

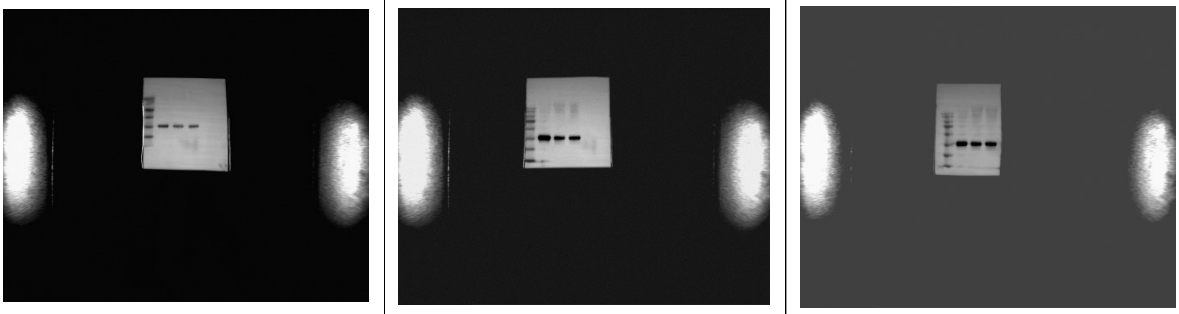

α-SMA	Sham	1	46895.279	1	49543.773	1	52768.108		2.007127046	2.527944853	1.055837266	1.863636388	1	1	1		1	0.400544838	
	ASDH	2	33467.158	2	42253.794	2	32289.551		1.492074903	1.194286594	0.791187131		0.800625547	0.6408367	0.424539431		0.622000559	0.188749283	0.213323968
	ASDH-DC	3	32069.43	3	46146.794	3	31211.865		0.957633505	1.289254708	0.793267218		0.513852118	0.6917952	0.425655575		0.543767631	0.135568354	

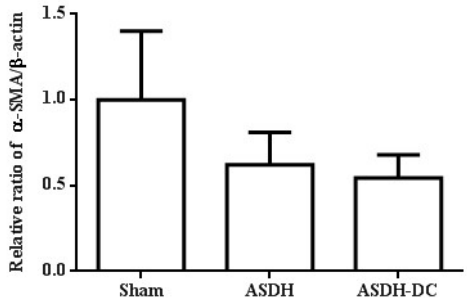

3.3 NG2

Sample1	Sample2	Sample3
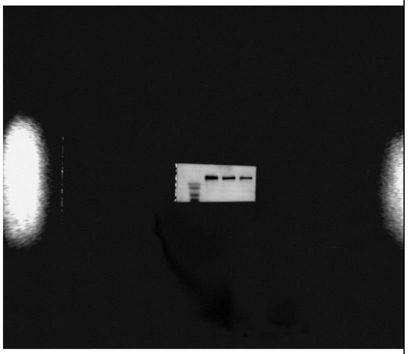	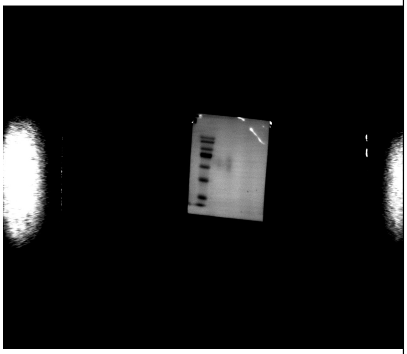	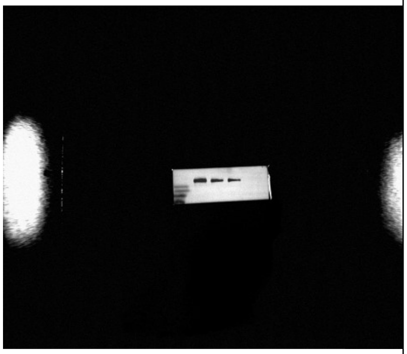

NG2	Sham	1	38675.785	1	44796.229	1	48523.3		1.655331107	2.285703928	0.970902887	1.63731264	1	1	1		1	0.401625002	
	ASDH	2	24843.2	2	33404.714	2	33970.693		1.107590768	0.944170886	0.832379959		0.676468709	0.576658887	0.508381807		0.587169801	0.08453497	0.156435217
	ASDH-DC	3	17674.179	3	26620.744	3	28086.744		0.527773209	0.743733563	0.713840499		0.322341131	0.454240409	0.435983013		0.404188185	0.071467043	

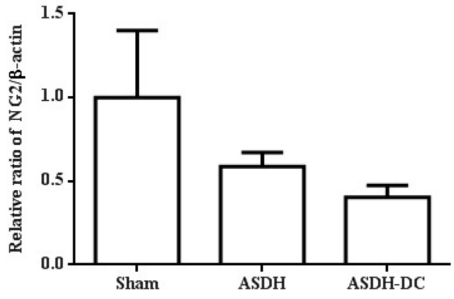

4 Image Clip

1	Sham	ASDH	ASDH-DC
β-actin	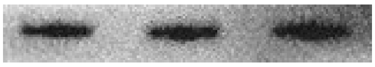		

α-SMA	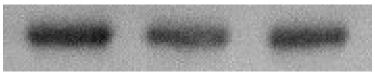
PDGFRβ	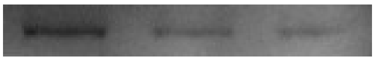
NG2	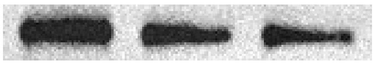

5 ELISA data

Table format: Grouped		Group A Sham			Group B ASDH			Group C ASDH-DC		
		Mean	SD	N	Mean	SD	N	Mean	SD	N
1	TNF-α	75.852467	14.183452	3	128.747235	17.968088	6	157.364751	18.542149	6
2	IL-1β	31.135647	7.332458	3	63.245127	6.213659	6	74.586937	8.687954	6
3	IL-6	72.157869	10.357845	3	148.654251	11.234852	6	152.634812	9.248375	6
4	IFN-γ	57.288452	10.851438	3	114.256743	12.450123	6	121.425935	10.240350	6

Table format: Grouped		Group A Sham			Group B ASDH			Group C ASDH-DC		
		Mean	SD	N	Mean	SD	N	Mean	SD	N
1	ROS	228.152648	26.548910	3	389.217560	40.245175	6	405.256802	42.105793	6
2	RNS	102.068134	30.240678	3	215.324086	31.240182	6	224.256720	29.251504	6

6 Physiological data

6.1 MABP and ICP

Table format: XY		X time(min)	Group A MABP-sham			Group B ICP-sham			Group C MABP-ASDH			Group D ICP-ASDH		
		X	Mean	SD	N	Mean	SD	N	Mean	SD	N	Mean	SD	N
1	baseline		90.833	4.284	12	4.225	0.505	12	91.858	3.951	12	4.175	0.594	12
2	modeling		88.717	4.317	12	4.400	0.433	12	110.833	8.515	12	46.225	7.507	12
3	5		88.208	2.459	12	4.050	0.518	12	99.142	3.297	12	28.658	5.375	12
4	10		88.825	3.267	12	4.150	0.480	12	92.533	3.575	12	18.900	5.978	12
5	20		89.267	3.181	12	3.675	0.657	12	91.750	3.355	12	17.900	3.732	12
6	30		88.900	3.161	12	4.108	0.565	12	89.300	3.234	12	17.792	4.741	12
7	60		89.442	3.749	12	4.092	0.562	12	90.717	3.311	12	18.575	3.631	12
8	90		89.808	1.656	12	4.275	0.535	12	92.625	2.659	12	22.433	5.152	12
9	120		87.967	3.067	12	4.608	0.619	12	94.560	3.029	12	24.608	4.469	12
10	150		89.283	2.468	12	4.692	0.446	12	94.225	5.031	12	26.392	4.003	12
11	180		90.350	3.028	12	4.492	0.578	12	95.667	4.026	12	28.683	3.309	12
12	210		87.225	1.926	12	4.525	0.469	12	96.042	3.760	12	28.350	4.056	12
13	240		89.625	3.356	12	4.417	0.374	12	95.517	4.169	12	28.700	4.555	12

6.2 rCBF

Table format: XY		X time(min)	Group A Artery-sham			Group B Vein-sham			Group C Capillary-sham		
		X	Mean	SD	N	Mean	SD	N	Mean	SD	N
1	baseline		100.0000	0.0000	12	100.0000	0.0000	12	100.0000	0.0000	12
2	DC		111.0792	6.6378	12	109.0850	4.4569	12	109.4775	5.9960	12
3	5		124.2808	6.9398	12	120.3617	7.5061	12	123.8600	4.7768	12
4	10		125.1233	6.0156	12	126.1767	7.6009	12	124.1783	7.5717	12
5	15		128.8083	5.8657	12	129.4125	6.8007	12	127.8933	7.2118	12
6	20		125.1500	7.6777	12	127.8025	6.5454	12	126.1283	6.3045	12
7	25		123.9458	5.8625	12	125.6092	5.4343	12	123.9300	5.3469	12
8	30		122.4758	5.0943	12	124.9750	4.6351	12	123.1525	5.0128	12

Table format: XY		X	Group A			Group B			Group C		
		time(min)	Artery-ASDH			Vein-ASDH			Capillary-ASDH		
		X	Mean	SD	N	Mean	SD	N	Mean	SD	N
1	baseline		100.0000	0.0000	12	100.0000	0.0000	12	100.0000	0.0000	12
2	DC		63.8900	9.5155	12	49.3083	9.6542	12	50.7117	8.0434	12
3	5		69.0483	10.6924	12	50.3917	8.8741	12	52.4308	7.8556	12
4	10		74.3308	12.7850	12	50.9158	9.5518	12	52.1050	8.0702	12
5	15		74.2317	14.0949	12	51.9258	10.5475	12	55.3492	7.7754	12
6	20		69.0967	12.0468	12	53.7525	8.4626	12	55.3317	8.0293	12
7	25		63.9575	10.1436	12	51.4500	9.8679	12	54.6808	8.2553	12
8	30		61.3658	9.6289	12	52.0508	7.7592	12	54.8067	7.0201	12

Western-blot reports (PART TWO)

1. Experimental instruments and reagents

	ITEM NO	Manufacturer or brand
PVDF（0.22um）	ISEQ00010	Millipore
β-actin antibody	ab8226	abcam
Lamin B1 Polyclonal antibody	12987-1-AP	Proteintech
Phospho-NF-κB p65 (Ser536) (93H1) Rabbit mAb	3033	CST
NF-κB p65 (D14E12) XP® Rabbit mAb	8242	CST
Anti-NG2 antibody	Ab275024	abcam
NOX2 Polyclonal antibody	19013-1-AP	Proteintech
NOX4 Polyclonal antibody	14347-1-AP	Proteintech
Anti-alpha smooth muscle Actin antibody	Ab124964	abcam
Neurotrophin 3 Polyclonal antibody	18084-1-AP	Proteintech
Anti-Occludin antibody	Ab216327	abcam
ZO-1 antibody	21773-1-ap	Proteintech
Claudin 5 Polyclonal antibody	29767-1-AP	Proteintech
AQP4 antibody	16473-1-AP	Proteintech
	ITEM NO	Manufacturer or brand

β-actin antibody	ab8226	abcam
Lamin B1 Polyclonal antibody	12987-1-AP	Proteintech
Phospho-NF-κB p65 (Ser536) (93H1) Rabbit mAb	3033	CST
NF-κB p65 (D14E12) XP® Rabbit mAb	8242	CST
Anti-NG2 antibody	Ab275024	abcam
NOX2 Polyclonal antibody	19013-1-AP	Proteintech
NOX4 Polyclonal antibody	14347-1-AP	Proteintech
Anti-alpha smooth muscle Actin antibody	Ab124964	abcam
Neurotrophin 3 Polyclonal antibody	18084-1-AP	Proteintech
Anti-Occludin antibody	Ab216327	abcam
ZO-1 antibody	21773-1-ap	Proteintech
Claudin 5 Polyclonal antibody	29767-1-AP	Proteintech
AQP4 antibody	16473-1-AP	Proteintech

2. Experimental steps

As shown in Part I.

3. Experimental results

3.1.1 β-actin

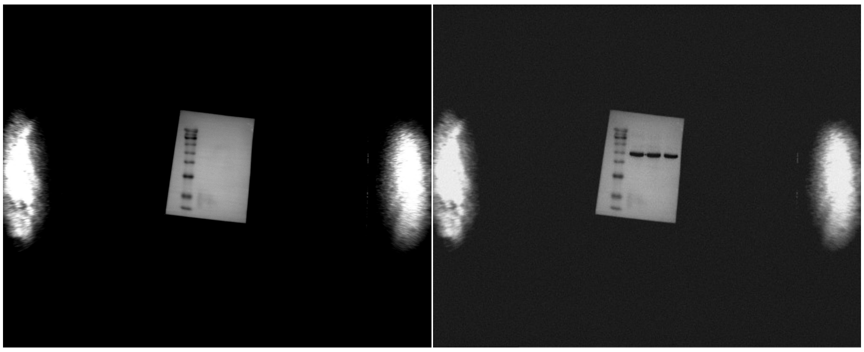

actin							
	sham	1	53164.744	1	51769.815	1	50237.007
	ASDH-DC	2	55224.057	2	53195.35	2	50655.522
	IFX	3	50968.957	3	49937.543	3	48805.836

3.1.2 NF-kB p65 cytoplasmic protein

Sample1	Sample2	Sample3
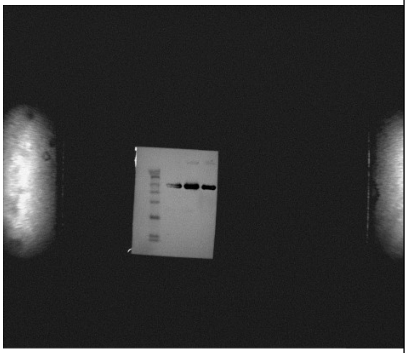	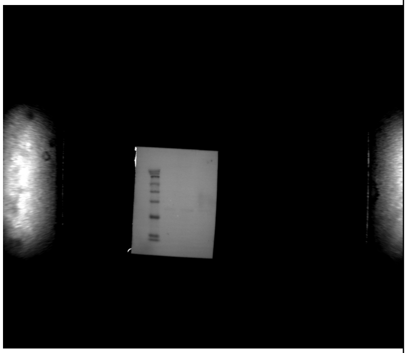	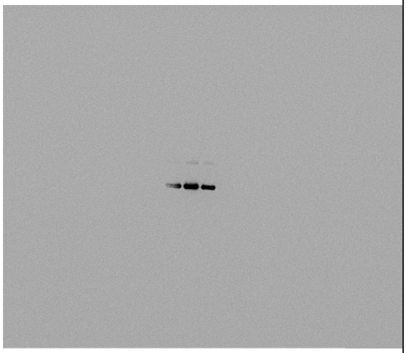

NF-kB p65 cytoplasmic protein																	AVG	SD	
	sham	1	22691.472	1	23816.836	1	24952.078		0.426814281	0.460052562	0.496687193	0.461184679	1	1	1		1	0.075783547	
	ASDH-DC	2	53247.664	2	54468.078	2	55694.664		0.964211376	1.02392555	1.099478631		2.090727253	2.220207213	2.384031132		2.231655199	0.146986678	0.000208261
	IFX	3	42596.664	3	42682.765	3	43908.057		0.835737408	0.854722969	0.899647677		1.812153453	1.85332039	1.950731928		1.87206859	0.071166142	0.018878851

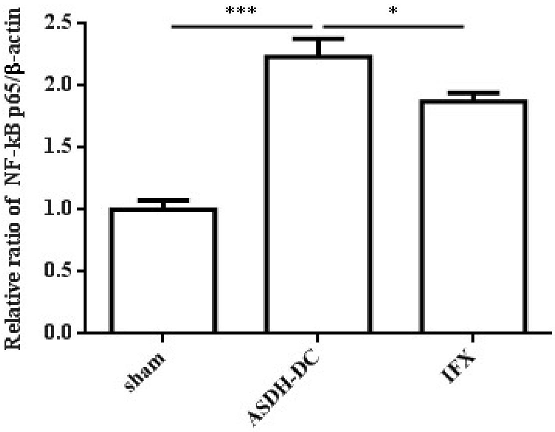

3.1.3 p-NF-kB p65 cytoplasmic protein

Sample1	Sample2	Sample3

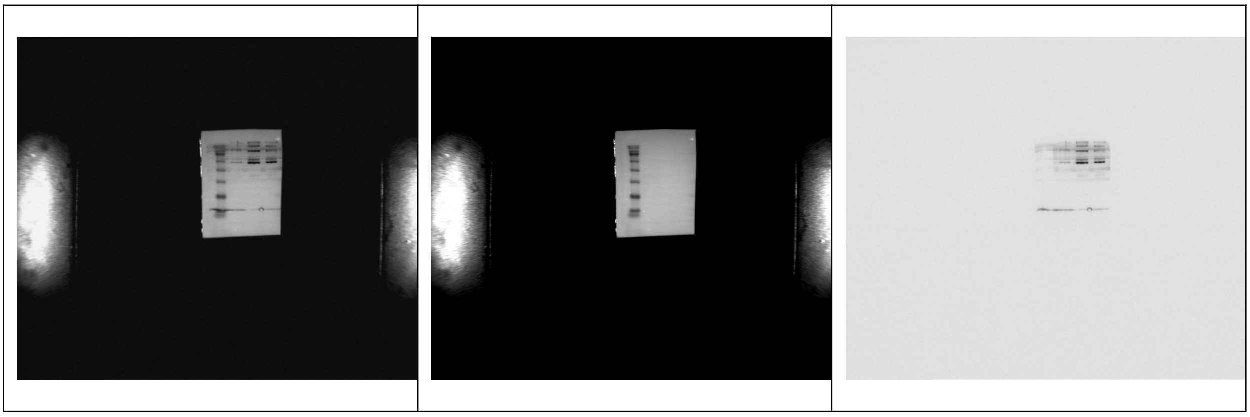

NF-kB p65 cytoplasmic protein																	AVG	SD	
	sham	1	22691.472	1	23816.836	1	24952.078		0.426814281	0.460052562	0.496687193	0.461184679	1	1	1		1	0.075783547	
	ASDH-DC	2	53247.664	2	54468.078	2	55694.664		0.964211376	1.02392555	1.099478631		2.090727253	2.220207213	2.384031132		2.231655199	0.146986678	0.000208261
	IFX	3	42596.664	3	42682.765	3	43908.057		0.835737408	0.854722969	0.899647677		1.812153453	1.85332039	1.950731928		1.87206859	0.071166142	0.018878851

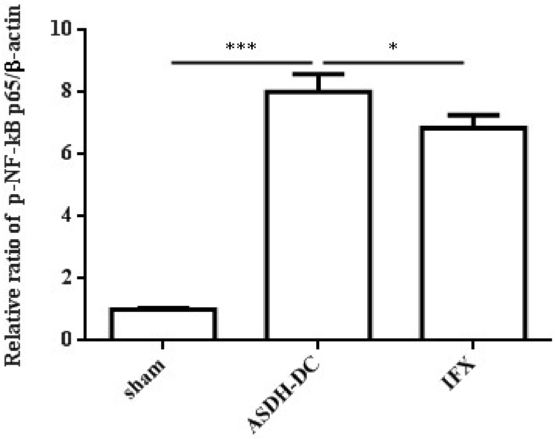

3.2.1 Lamin B1

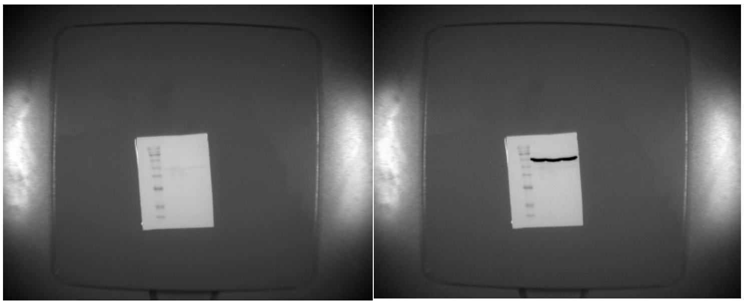

Lamin B1							
	sham	1	57368.472	1	55385.643	1	53167.108
	ASDH-DC	2	58638.522	2	57030.401	2	56167.401
	IFX	3	59443.693	3	57040.643	3	54115.179

3.2.2 NF-kB p65 nuclear protein

Sample1	Sample2	Sample3
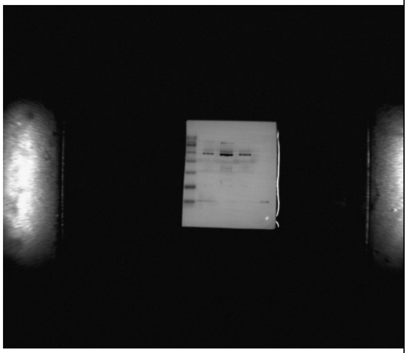	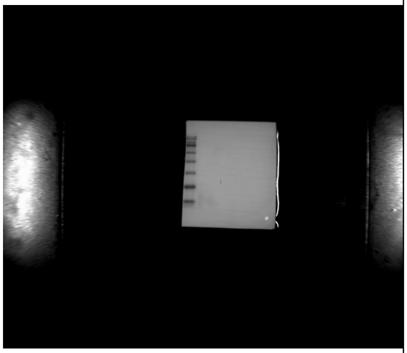	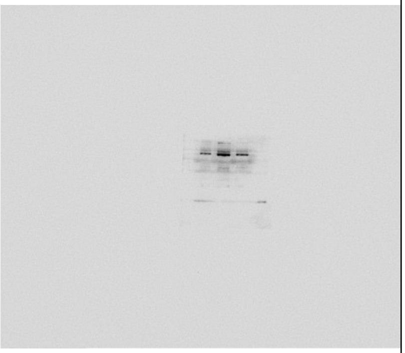

NF-kB p65 nuclear protein																			
	sham	1	15900.359	1	16542.329	1	17618.593		0.277161975	0.298675399	0.331381444	0.302406272	1	1	1		1	0.09028119	
	ASDH-DC	2	48635.22	2	50088.806	2	51359.756		0.829407331	0.87828255	0.914405066		2.74269222	2.90431327	3.023763556		2.890256349	0.141061943	4.03764E-05
	IFX	3	23983.756	3	24881.785	3	25527.158		0.403470155	0.43621151	0.471718998		1.334199027	1.442468459	1.559884965		1.445517484	0.112873859	0.0001575

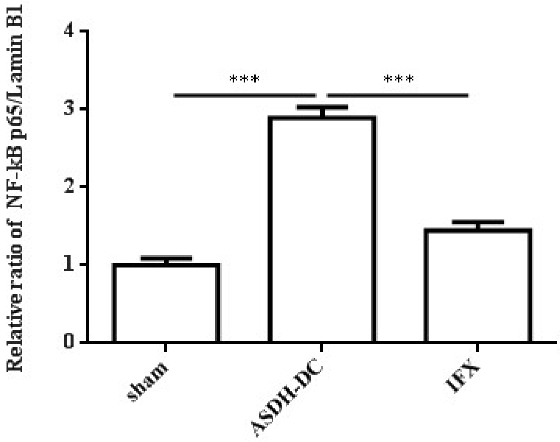

3.3.1 β-actin

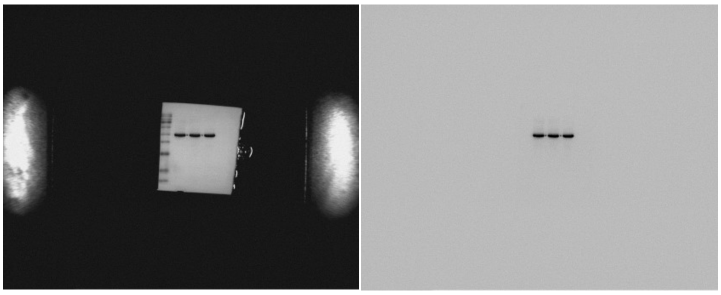

actin							
	sham	1	50573.342	1	48802.078	1	47012.057
	vehicle	2	50419.128	2	50113.421	2	49111.007
	PBN	3	48974.785	3	47185.765	3	46104.38

3.3.2 NOX2

Sample1	Sample2	Sample3
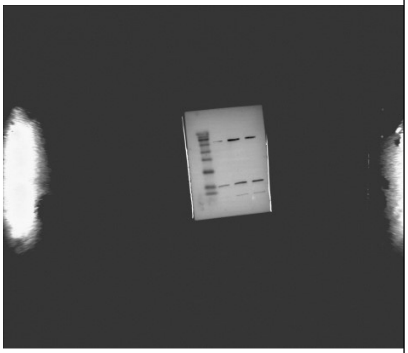	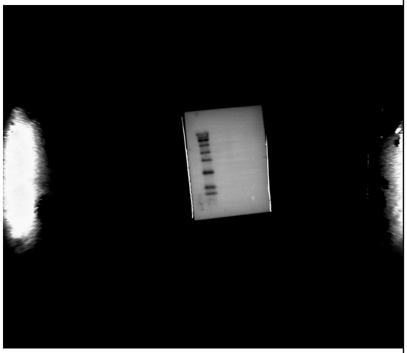	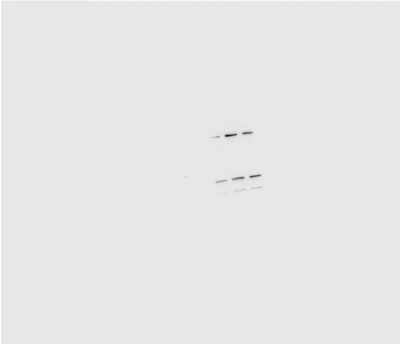

NOX2															
	sham	1	5892.702	1	6007.53	1	6030.652		0.116517947	0.123099881	0.128278837	0.122632222	1	1	1
	vehicle	2	38284.421	2	39726.321	2	41496.999		0.759323346	0.79272818	0.844963309		6.191874657	6.464273155	6.890222624
	PBN	3	32407.472	3	33214.451	3	33616.48		0.661717494	0.703908287	0.729138533		5.395951277	5.739994564	5.945733691

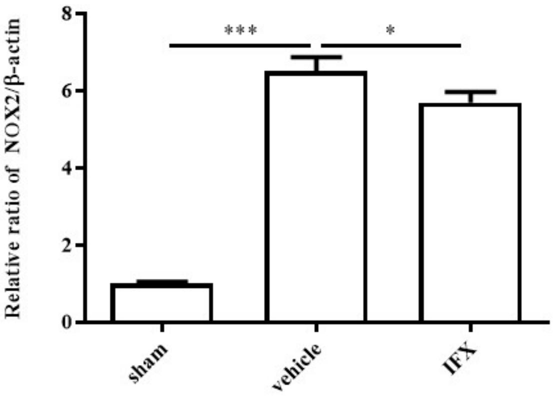

3.3.3 NOX4

Sample1	Sample2	Sample3
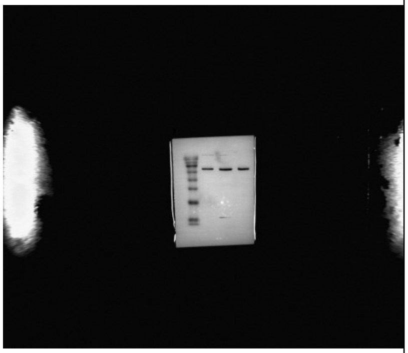	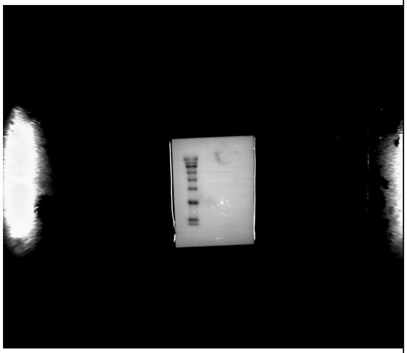	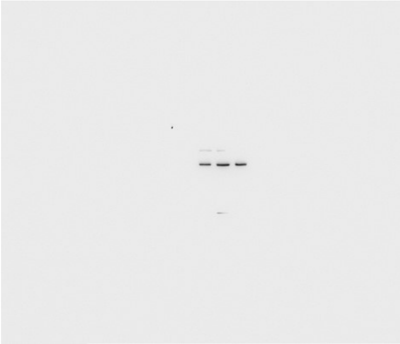

NOX4															
	sham	1	28806.966	1	28969.572	1	28933.279		0.569607719	0.593613493	0.6154438	0.592888338	1	1	1
	vehicle	2	50675.886	2	51311.492	2	53539.049		1.005092472	1.023907188	1.090163942		1.6952475	1.726981497	1.838733996
	PBN	3	41280.078	3	42560.472	3	43512.329		0.842884313	0.901976942	0.943778639		1.421657772	1.521326841	1.591832018

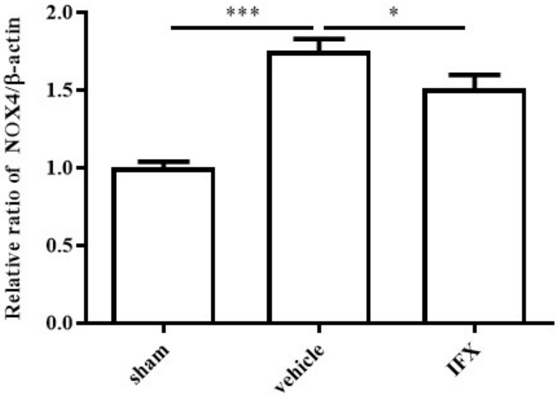

3.4.1 β-actin

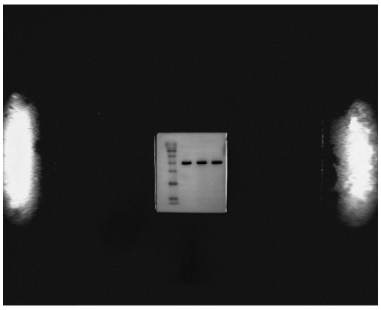

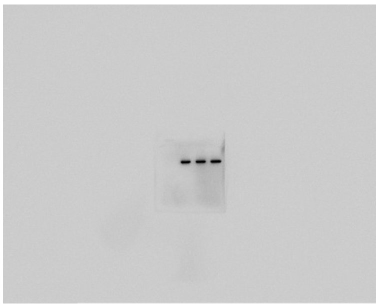

actin							
	sham	1	39386.744	1	37791.137	1	36578.572
	vehicle	2	48943.756	2	46145.563	2	42940.886
	IFX	3	54225.229	3	51472.128	3	48972.22

3.4.2 α-SMA

Sample1	Sample2	Sample3

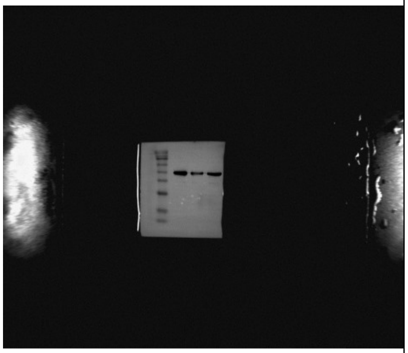	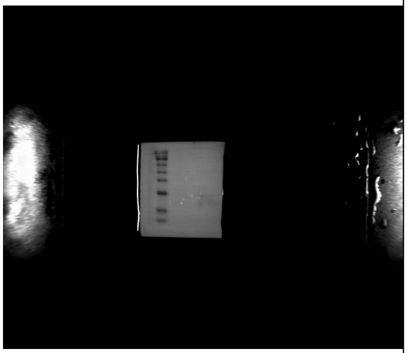	

a-SMA																	AVG	SD	
	sham	1	48910.279	1	49265.593	1	49457.25		1.241795438	1.303628229	1.35208258	1.299168749	1	1	1		1	0.042549242	
	vehicle	2	26932.472	2	26842.522	2	27529.886		0.55027391	0.581692372	0.641111271		0.423558457	0.447741968	0.493478058		0.454926161	0.035509113	6.96354E-05
	IFX	3	45789.836	3	46467.886	3	46568.451		0.844437854	0.902777635	0.950915662		0.649983195	0.694888663	0.731941607		0.692271155	0.041041855	0.001628581

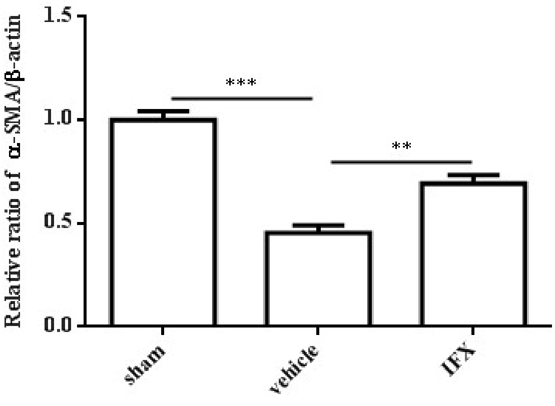

3.4.3 NG2

Sample1	Sample2	Sample3
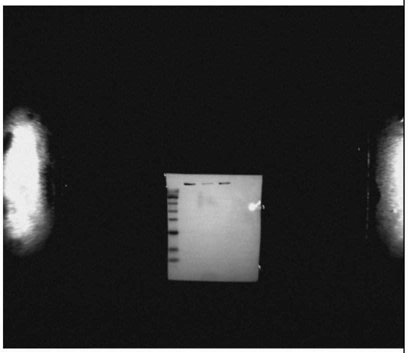	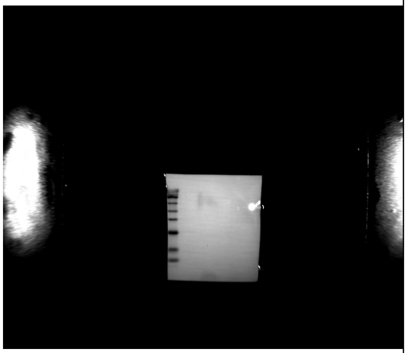	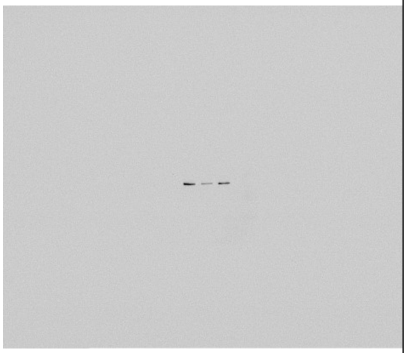

NG2																			
	sham	1	35450.208	1	36268.472	1	37094.321		0.900054292	0.95970841	1.014099758	0.957954153	1	1	1		1	0.059546657	
	vehicle	2	10600.723	2	11741.35	2	10918.501		0.216589896	0.254441581	0.254268182		0.226096306	0.265609351	0.265428341		0.252377999	0.022760794	3.46795E-05
	IFX	3	30461.635	3	30530.635	3	30256.635		0.561761297	0.593148879	0.61783262		0.586417727	0.61918295	0.644950093		0.616850257	0.029335824	7.01787E-05

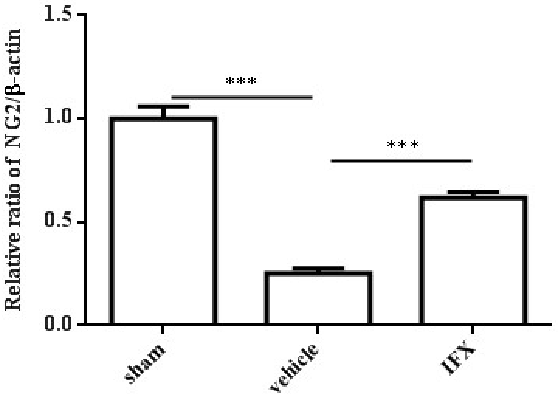

3.4.4 3-NT

Sample1	Sample2	Sample3
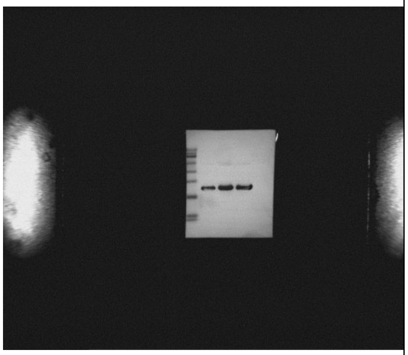	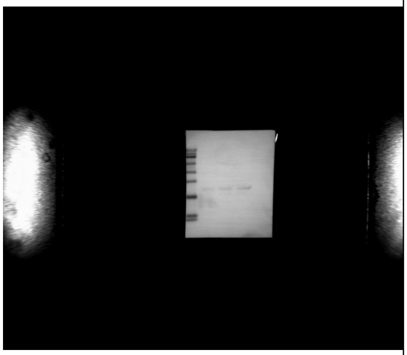	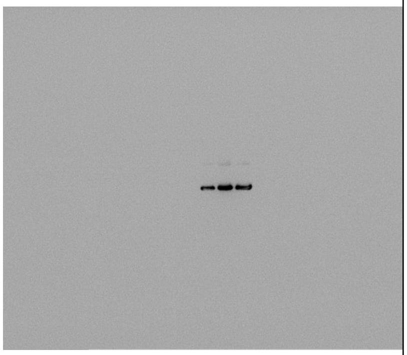

3-NT																			
	sham	1	31077.35	1	31324.007	1	31782.492		0.789030695	0.82887178	0.868882798	0.828928425	1	1	1		1	0.048165898	
	vehicle	2	55704.179	2	55010.593	2	56144.179		1.138126363	1.192110128	1.307476026		1.373009212	1.438133972	1.577308713		1.462817299	0.104362453	0.002222665
	IFX	3	48416.049	3	48871.978	3	49271.907		0.892869424	0.949484311	1.006119531		1.077136937	1.145435822	1.213759235		1.145443998	0.068311149	0.011626598

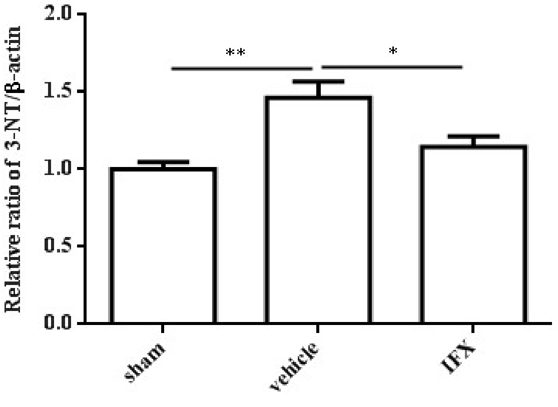

3.5.1 β-actin

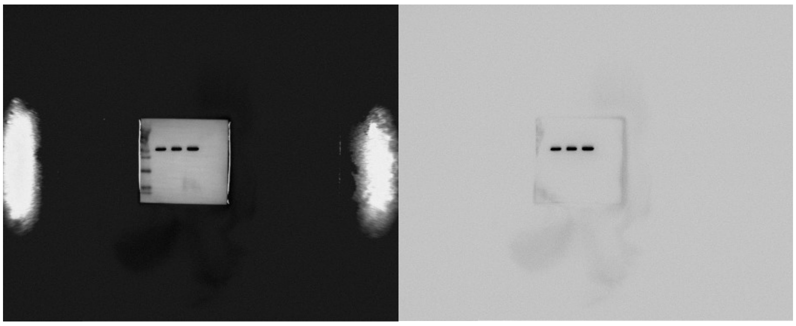

actin							
	Sham	1	42369.735	1	40137.522	1	38596.501
	ASDH	2	42919.622	2	41599.057	2	39706.765
	ASDH-DC	3	47700.3	3	45306.543	3	43996.492

3.5.2 occludin

**Sample1**	**Sample2**	**Sample3**
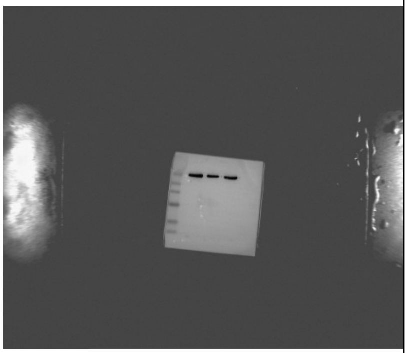	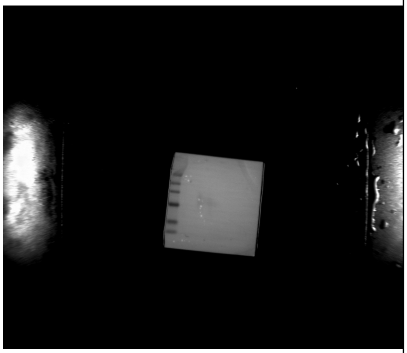	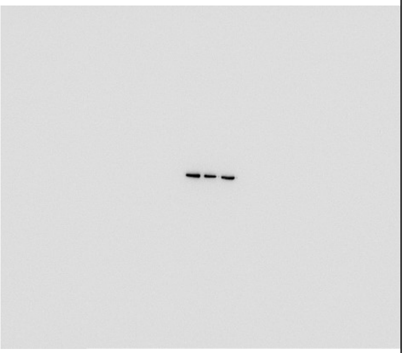

occludin																	AVG	SD	
	Sham	1	50150.765	1	50750.078	1	51172.978		1.183645944	1.264404863	1.325845003	1.25796527	1	1	1		1	0.056693068	
	ASDH	2	34515.622	2	34329.572	2	34571.057		0.804192125	0.825248803	0.870659118		0.639280069	0.656018749	0.692116976		0.662471931	0.027003099	0.00074079
	ASDH-DC	3	43287.551	3	42750.693	3	45286.907		0.907490121	0.943587618	1.029329952		0.72139521	0.750090357	0.818249896		0.763245154	0.049749312	0.036804913

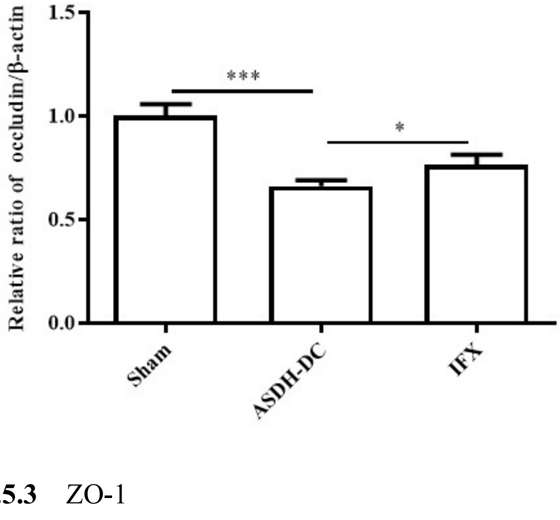

3.5.3 ZO-1

Sample1	Sample2	Sample3
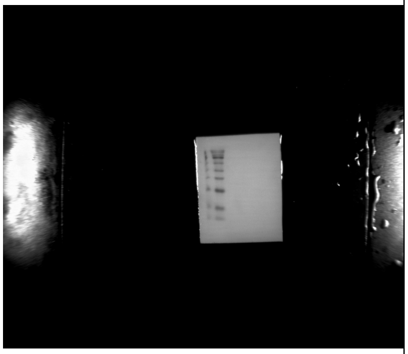	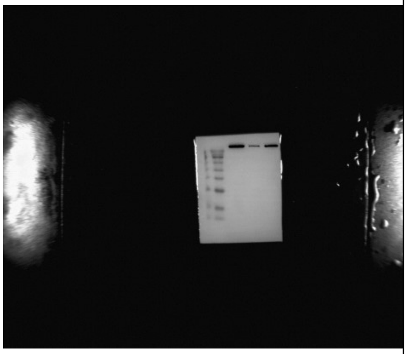	

ZO-1																			
	Sham	1	58188.614	1	58838.927	1	59897.463		1.373353267	1.465933223	1.551888421	1.463724971	1	1	1		1	0.061000571	
	ASDH	2	10995.622	2	11349.066	2	12290.409		0.256191026	0.272820271	0.309529346		0.175026751	0.186387659	0.211466875		0.190960429	0.018645464	2.5408E-05
	ASDH-DC	3	42644.25	3	43248.957	3	43275.057		0.894003811	0.954585235	0.983602443		0.610773082	0.652161611	0.671985832		0.644973508	0.031233025	2.70824E-05

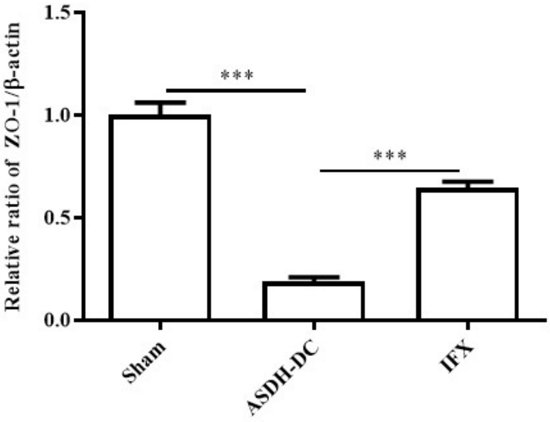

3.5.4 claudin5

Sample1	Sample2	Sample3
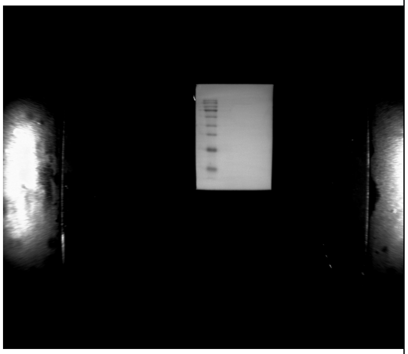	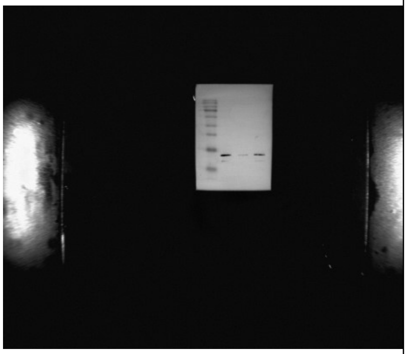	

Claudin5																			
	Sham	1	35125.329	1	35426.693	1	35609.765		0.829019322	0.882632789	0.922616405	0.878089506	1	1	1		1	0.0534839	
	ASDH	2	6793.894	2	7329.208	2	7787.693		0.158293426	0.176186878	0.19613013		0.180270263	0.200647972	0.223360067		0.2014261	0.021555438	1.79167E-05
	ASDH-DC	3	30674.057	3	30124.744	3	29794.844		0.643057947	0.664909349	0.677209538		0.732337584	0.757222748	0.771230647		0.753596993	0.019698405	5.18175E-06

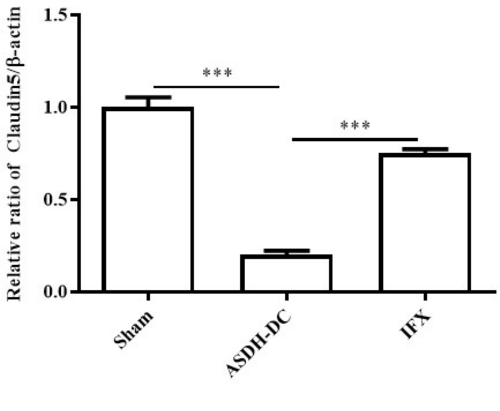

3.5.5 AQP4

**Sample1**	**Sample2**	**Sample3**
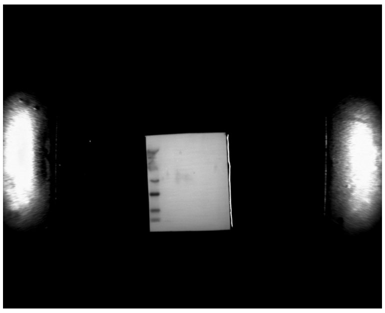	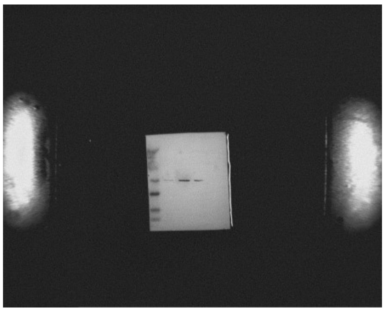	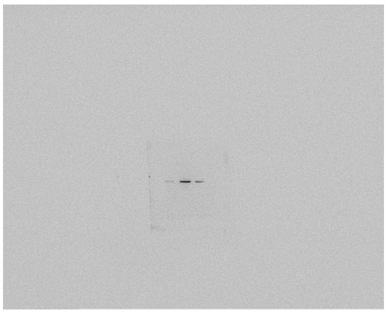

AQP4																			
	Sham	1	9537.622	1	9643.551	1	9814.966		0.225104594	0.24026274	0.254296782	0.239888039	1	1	1		1	0.060860478	
	ASDH	2	44413.049	2	45383.362	2	46253.919		1.034795903	1.090970932	1.164887621		4.313661944	4.547833809	4.855963754		4.572486503	0.271990128	2.43682E-05
	ASDH-DC	3	16747.258	3	17532.986	3	18675.714		0.351093347	0.386985738	0.424481888		1.463571712	1.613193141	1.769500016		1.615421623	0.152976326	8.06745E-05

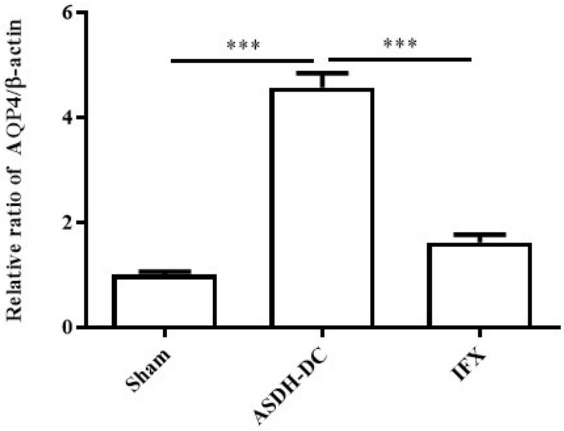

4 Image Clip

	sham	ASDH-DC	IFX
β-actin	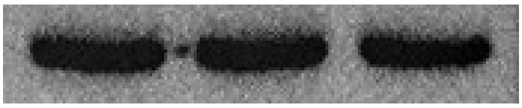		
NF-kB p65	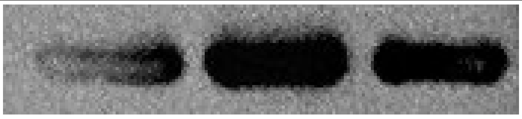		
p-NF-kB p65	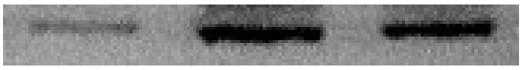		
Lamin B1	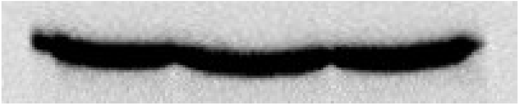		
NF-kB p65	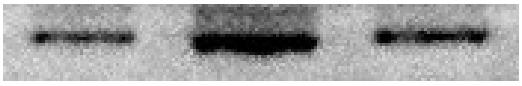		

	sham	vehicle	IFX
β-actin	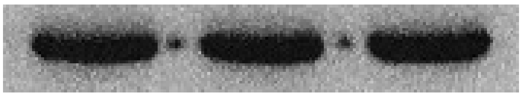		
NOX2	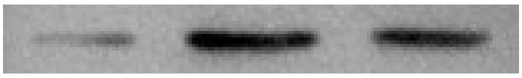		
NOX4	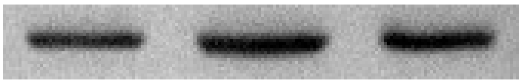		

	sham	vehicle	IFX
β-actin	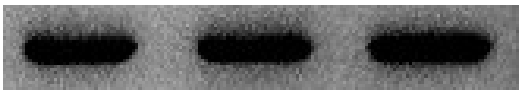		
a-SMA	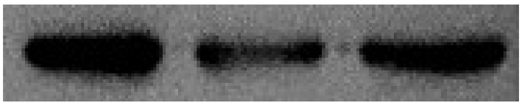		
NG2	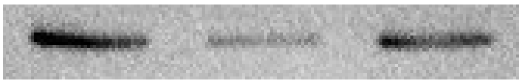		
3-NT	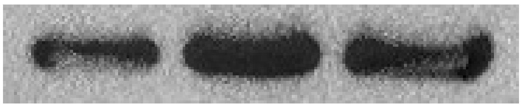		
	sham	vehicle	IFX
β-actin	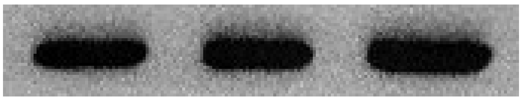		
occudin	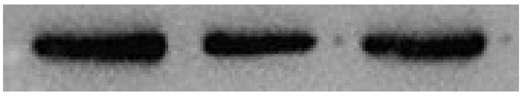		
ZO-1	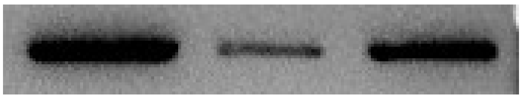		
Claudin5	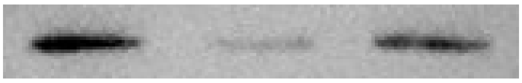		
AQP4	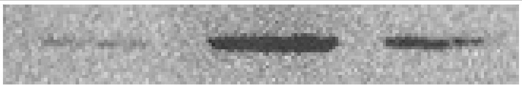		

5 ELISA

Table format: Grouped		Group A			Group B			Group C		
		Sham			vehicle			IFX		
		Mean	SD	N	Mean	SD	N	Mean	SD	N
1	TNF-α	75.852467	14.183452	3	157.364751	18.542149	6	104.235642	15.475214	6
2	IL-1β	31.135647	7.332458	3	74.586937	8.687954	6	60.245863	6.024108	6
3	IL-6	72.157869	10.357845	3	152.634812	9.248375	6	110.021045	10.258967	6
4	IFN-γ	57.288452	10.851438	3	121.425935	10.240350	6	79.102456	10.324792	6

Table format: Grouped		Group A			Group B			Group C		
		Sham			vehicle			IFX		
		Mean	SD	N	Mean	SD	N	Mean	SD	N
1	ROS	228.152648	26.548910	3	408.321502	41.254632	6	312.012342	39.546325	6
2	RNS	104.068134	28.364521	3	225.324086	29.854679	6	184.256720	26.458921	6
3	MDA	136.217536	22.124586	3	289.234562	32.785240	6	193.149602	25.457215	6

6 Physiological data

6.1 Aartery CBF

Table format: XY		X	Group A			Group B			Group C		
		time(min)	sham			vehicle			IFX		
		X	Mean	SD	N	Mean	SD	N	Mean	SD	N
1	baseline		100.0000	0.0000	12	100.00000	0.00000	12	100.0000	0.0000	12
2	DC		116.0792	6.6378	12	63.74120	9.01246	12	65.5425	12.0434	12
3	30min		122.8083	5.8657	12	62.48320	12.12548	12	70.4521	10.8556	12
4	3h		110.5589	5.8215	12	59.31450	7.57648	12	70.6589	6.0702	12
5	6h		113.8083	6.0314	12	57.45210	8.54723	12	72.2145	7.7754	12
6	9h		111.1500	5.6789	12	57.84200	9.08542	12	71.5421	8.0293	12
7	12h		113.9458	6.3459	12	57.14210	7.54215	12	71.8458	8.2553	12

6.2 Vein CBF

Table format: XY		X	Group A			Group B			Group C		
		time(min)	sham			vehicle			IFX		
		X	Mean	SD	N	Mean	SD	N	Mean	SD	N
1	baseline		100.0000	0.0000	6	100.00000	0.00000	12	100.0000	0.0000	12
2	DC		109.2562	5.2131	6	48.98670	6.24578	12	55.5462	10.2453	12
3	30min		122.3085	6.8569	6	52.25470	5.86216	12	60.3245	8.4575	12
4	3h		110.6582	6.2145	6	50.21450	6.65214	12	65.2142	6.5214	12
5	6h		112.5468	6.0246	6	49.24530	6.55142	12	68.4512	7.1275	12
6	9h		110.5462	7.0014	6	49.92580	7.01235	12	67.2531	6.9514	12
7	12h		112.2546	6.2457	6	48.75250	6.98512	12	67.5462	8.0215	12

6.3 Capillary CBF

Table format: XY		X	Group A			Group B			Group C		
		time(min)	sham			vehicle			IFX		
		X	Mean	SD	N	Mean	SD	N	Mean	SD	N
1	baseline		100.0000	0.0000	6	100.00000	0.00000	12	100.00000	0.00000	12
2	DC		112.5645	4.3586	6	49.21450	6.24512	12	52.54720	9.65879	12
3	30min		122.5462	5.6521	6	54.21450	6.01485	12	60.52415	12.25435	12
4	3h		113.0214	5.9654	6	49.25470	5.96324	12	69.25360	9.54253	12
5	6h		114.2145	6.3246	6	50.35670	7.21450	12	70.24520	8.65421	12
6	9h		110.5246	6.3246	6	48.62450	6.56324	12	69.54680	9.01254	12
7	12h		112.9456	6.6354	6	48.65720	7.10245	12	70.95360	8.23556	12

6.4 rCBF of IFX group

Table format: XY		X	Group A			Group B			Group C		
		time(min)	Artery-ASDH			Vein-ASDH			Capillary-ASDH		
		X	Mean	SD	N	Mean	SD	N	Mean	SD	N
1	baseline		100.0000	0.0000	12	100.0000	0.0000	12	100.00000	0.00000	12
2	DC		65.5425	12.0434	12	50.5462	10.2453	12	52.54720	9.65879	12
3	5		72.4521	10.8556	12	56.3245	8.4575	12	63.02415	12.25435	12
4	10		76.6589	6.0702	12	58.2142	6.5214	12	66.85360	9.54253	12
5	15		76.2145	7.7754	12	66.4512	7.1275	12	68.24520	8.65421	12
6	20		74.5421	8.0293	12	67.2531	6.9514	12	69.54680	9.01254	12
7	25		72.8458	8.2553	12	67.5462	8.0215	12	68.95360	8.23556	12
8	30		70.4521	6.2565	12	66.3245	7.6582	12	67.52415	7.25468	12

7 IF and IHC-P

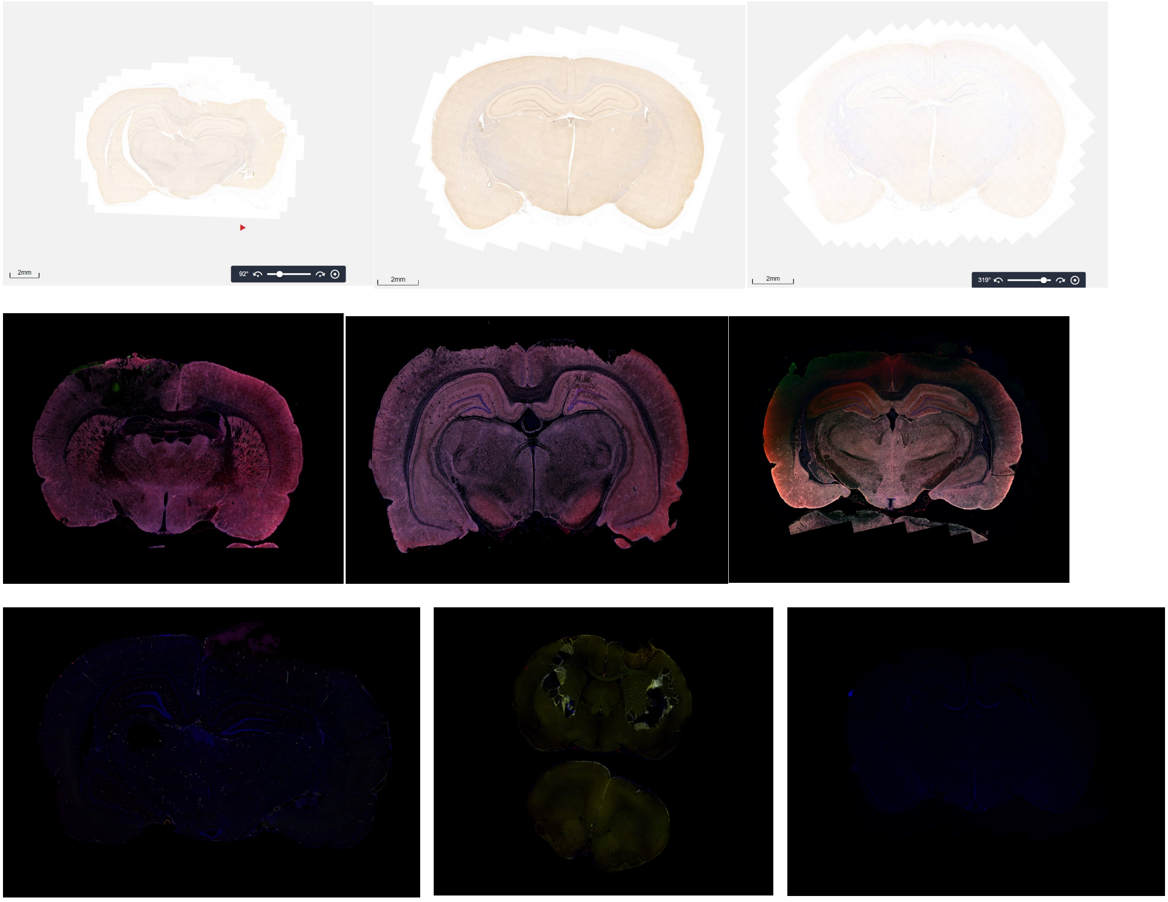
2mm
92°
2mm
2mm
319°
